# Global Gene Expression and Systems Biology Analysis of Bovine Monocyte-Derived Macrophages in Response to *In Vitro* Challenge with *Mycobacterium bovis*

**DOI:** 10.1371/journal.pone.0032034

**Published:** 2012-02-22

**Authors:** David A. Magee, Maria Taraktsoglou, Kate E. Killick, Nicolas C. Nalpas, John A. Browne, Stephen D. E. Park, Kevin M. Conlon, David J. Lynn, Karsten Hokamp, Stephen V. Gordon, Eamonn Gormley, David E. MacHugh

**Affiliations:** 1 Animal Genomics Laboratory, UCD School of Agriculture and Food Science, University College Dublin, Belfield, Dublin, Ireland; 2 UCD School of Veterinary Medicine, University College Dublin, Belfield, Dublin, Ireland; 3 Animal Bioscience Centre, Teagasc, Grange, Dunsany, County Meath, Ireland; 4 Smurfit Institute of Genetics, Trinity College Dublin, Trinity College, Dublin, Ireland; 5 UCD Conway Institute of Biomolecular and Biomedical Research, University College Dublin, Belfield, Dublin, Ireland; 6 Tuberculosis Diagnostics and Immunology Research Centre, UCD School of Veterinary Medicine, University College Dublin, Belfield, Dublin, Ireland; Institut Pasteur, France

## Abstract

**Background:**

*Mycobacterium bovis*, the causative agent of bovine tuberculosis, is a major cause of mortality in global cattle populations. Macrophages are among the first cell types to encounter *M. bovis* following exposure and the response elicited by these cells is pivotal in determining the outcome of infection. Here, a functional genomics approach was undertaken to investigate global gene expression profiles in bovine monocyte-derived macrophages (MDM) purified from seven age-matched non-related females, in response to *in vitro* challenge with *M. bovis* (multiplicity of infection 2∶1). Total cellular RNA was extracted from non-challenged control and *M. bovis*-challenged MDM for all animals at intervals of 2 hours, 6 hours and 24 hours post-challenge and prepared for global gene expression analysis using the Affymetrix® GeneChip® Bovine Genome Array.

**Results:**

Comparison of *M. bovis*-challenged MDM gene expression profiles with those from the non-challenged MDM controls at each time point identified 3,064 differentially expressed genes 2 hours post-challenge, with 4,451 and 5,267 differentially expressed genes detected at the 6 hour and 24 hour time points, respectively (adjusted *P*-value threshold ≤0.05). Notably, the number of downregulated genes exceeded the number of upregulated genes in the *M. bovis*-challenged MDM across all time points; however, the fold-change in expression for the upregulated genes was markedly higher than that for the downregulated genes. Systems analysis revealed enrichment for genes involved in: (1) the inflammatory response; (2) cell signalling pathways, including Toll-like receptors and intracellular pathogen recognition receptors; and (3) apoptosis.

**Conclusions:**

The increased number of downregulated genes is consistent with previous studies showing that *M. bovis* infection is associated with the repression of host gene expression. The results also support roles for MyD88-independent signalling and intracellular PRRs in mediating the host response to *M. bovis*.

## Introduction

Bovine tuberculosis (BTB), an infectious disease caused by the intracellular pathogen *Mycobacterium bovis*, continues to impact the welfare of domestic herds, despite the implementation of surveillance and control programmes in many countries. The aetiology and host immune response to *M. bovis* infection is similar to *M. tuberculosis* infection in humans [1], [2]. *M. bovis* is transmitted primarily via aerosolised respiratory secretions containing infectious bacilli, with the natural site of infection being the respiratory tract. Following initial exposure, the pathogen is encountered by host alveolar macrophages, which serve as key effector cells in activating the innate and adaptive immune responses required to determine the outcome of infection [3].

Macrophage recognition of mycobacteria occurs through the interaction of mycobacterial pathogen-associated molecular patterns (PAMPs) with host pathogen recognition receptors (PRRs), such as the Toll-like receptors (TLRs), expressed on the macrophage cell surface [4]. PRR activation induces signalling pathways resulting in the production of endogenous NF-κB-inducible cytokines that promote an adaptive immune response characterised by the release of proinflammatory interferon-gamma (IFN-γ) from T cells and natural killer (NK) cells [5], [6]. In turn, IFN-γ induces microbicidal activity in infected macrophages and enhances the expression of major histocompatibility complex (MHC) class I and II molecules necessary for the presentation of mycobacterial antigens on the macrophage surface to T cells [4], [5].

These molecular mechanisms culminate in the formation of granulomas―organised complexes of immune cells comprised of lymphocytes, non-infected macrophages and neutrophils that contain mycobacterial-infected macrophages and prevent the dissemination of bacilli to other organs and tissues―however, in most cases the pathogen is not eliminated by the host [4]. The survival of mycobacteria in host macrophages (within granulomas) is believed to be achieved through a diverse range of molecular mechanisms that subvert the host immune response. These include the prevention of host phagosome maturation, the inhibition of apoptosis in infected macrophages and the suppression of innate cell signalling pathways and cytokine production necessary to activate adaptive immune responses [7]–[9].

This persistence of mycobacteria can result in progression to active tuberculosis, particularly in cases where the immune response to infection is compromised [2], [4]. Furthermore, it has been proposed that mycobacteria can exploit several host defence mechanisms, such as cytokine-induced necrosis, resulting in immune pathology, dissemination of infection through the host and ultimately shedding of the pathogen from the host, thus maintaining the cycle of infection [10], [11]. Consequently, analysis of the macrophage transcriptome in response to mycobacterial infection may provide insight into the cellular mechanisms governing mycobacteria-macrophage interactions, enabling further understanding of how modulation of these pathways can result in pathology. In addition, identification of transcriptional markers of infection may lead to the development of novel diagnostics for BTB, providing new molecular tools for disease control and eradication [12].

The recent availability of a complete *Bos taurus* genome [13], coupled with the continuing development of high-throughput genomic technologies, have enabled analysis of the transcriptional changes induced in bovine macrophages following challenge with *M. bovis in vitro* [14], [15]. In the current study, purified monocyte-derived macrophages (MDM) from seven age-matched unrelated Holstein-Friesian females were used for *in vitro* challenge experiments with cultured *M. bovis* (multiplicity of infection [MOI] 2∶1). Total cellular RNA was extracted from challenged and non-challenged control MDM samples at intervals of 0 hours (*i.e.* pre-infection) and 2 hours, 6 hours and 24 hours post-challenge and prepared for global gene expression analyses using the high-density Affymetrix® GeneChip® Bovine Genome Array. This microarray contains 24,072 probe sets representing over 23,000 gene transcripts. Differentially expressed genes, identified through comparison of the gene expression profiles from the *M. bovis*-challenged MDM and the non-challenged control MDM at the 2 hour, 6 hour and 24 hour time points, were subsequently analysed with the Ingenuity® Pathway Analysis Knowledge Base (http://www.ingenuity.com) and used to reconstruct the macrophage cellular pathways underlying *M. bovis* infection. These data add a novel layer of information regarding the complex macrophage-specific molecular pathways elicited upon phagocytosis of *M. bovis* and the role these pathways play in establishing the host immune response to BTB.

## Materials and Methods

### Ethics statement

All animal procedures were carried out according to the provisions of the Irish Cruelty to Animals Act (Department of Health and Children licence number B100/3939) and ethical approval for the study was obtained from the UCD Animal Ethics Committee (protocol number AREC-P-07-25-MacHugh).

### Animals

Seven age-matched (three-year old) unrelated Holstein-Friesian females were used in this study. All animals were maintained under uniform housing conditions and nutritional regimens at the UCD Lyons Research Farm (Newcastle, County Kildare, Ireland). The animals were selected from an experimental herd without a recent history of bovine tuberculosis infection and all animals tested negative for the single intradermal tuberculin test (SICTT). These cattle were also negative for infection with *Brucella abortus*, *M. avium* subsp. *paratuberculosis*, *Salmonella* Typhimurium, bovine herpesvirus 1 (BHV-1) and bovine viral diarrhoea (BVD) virus.

### Monocyte extraction and culture of bovine MDM

For monocyte isolation, 300 ml of whole blood was collected in acid citrate dextrose buffer (Sigma-Aldrich Ireland Ltd., Dublin, Ireland) in sterile bottles. Blood was layered onto Accuspin™ tubes containing Histopaque® 1077 (Sigma-Aldrich Ireland Ltd., Dublin, Ireland), and following density gradient centrifugation (500 g for 20 minutes) performed at room temperature, peripheral blood mononuclear cells (PBMC) were collected. Contaminating red blood cells (RBC) were removed following resuspension and subsequent incubation of the PBMC in RBC lysis buffer (10 mM KHCO_3_, 150 mM NH_4_Cl, 0.1 mM EDTA pH 8.0) for 5 minutes at room temperature. After incubation, PBMC were washed twice with sterile phosphate-buffered saline (PBS; Invitrogen™, Life Technologies Corporation, Paisley, UK) before resuspending cells in PBS containing 1% bovine serum albumin (BSA; Sigma-Aldrich Ireland Ltd., Dublin, Ireland). Monocytes were then isolated using the MACS® protocol and MACS® MicroBeads conjugated to mouse anti-human CD14 antibodies (Miltenyi Biotec Ltd., Surrey, UK), which has been shown to be cross-reactive with bovine monocytes [16]. The MACS® protocol was performed according to the manufacturers' instructions.

The identity and purity of monocytes was confirmed by flow cytometry using an anti-CD14 fluorescein-labelled antibody (data not shown). This method has been previously shown by us to yield a purity of CD14^+^ cells ≥99% [17]. Purified monocytes were seeded at 1×10^6^ per well on 24-well tissue culture plates in RPMI 1640 medium (Invitrogen™, Life Technologies Corporation, Paisley, UK) containing 15% heat inactivated foetal calf serum (FCS; Sigma-Aldrich Ireland Ltd., Dublin, Ireland), 1% non-essential amino acids (NEAA; Sigma-Aldrich Ireland Ltd., Dublin, Ireland), gentamicin (5 µg/ml; Sigma-Aldrich Ireland Ltd., Dublin, Ireland) and incubated at 37°C, 5% CO_2_. Following 24 hours incubation (day one) the media was replaced with 1 ml fresh antibiotic-containing media to remove any non-adhered cells. On day three, media was replaced with 1 ml antibiotic-free culture media (RPMI 1640 medium containing 15% heat inactivated FCS and 1% NEAA only). To ensure that the same number of MDM were subjected to *M. bovis*-challenge, cells were dissociated on day five using 1× non-enzymatic cell dissociation solution (Sigma-Aldrich Ireland Ltd., Dublin, Ireland), counted and then re-seeded at 2×10^5^ cells per well in 24-well tissue culture plates (Sarstedt Ltd., County Wexford, Ireland) using antibiotic-free culture media. By day eight, 80–100% confluent monolayers of MDM were generated that displayed the characteristic macrophage morphology as confirmed by Giemsa staining (data not shown). On day eight, MDM were used for the *in vitro* challenge experiments with *M. bovis*.

### Culture of *M. bovis*

The *M. bovis* M2137 strain was used in this study. This strain is a clinical isolate from a tuberculous badger, isolated in 2001. The strain bears the SB0142 spoligotype, a strain type frequently isolated from badgers and cattle in Ireland. This isolate of *M. bovis* was supplied by Mr. Eamon Costello (Irish Department of Agriculture, Food and the Marine, Backweston Laboratory Campus, Celbridge, Co. Kildare).

Culturing of *M. bovis* was performed in a Biosafety Containment Level 3 (CL3) laboratory and conformed to the national guidelines on the use of Hazard Group 3 infectious organisms. *M. bovis* stocks for the *in vitro* MDM-challenge experiments were cultured in Middlebrook 7H9 medium (Difco™, Becton, Dickinson Ltd., Oxford, UK) containing 10% (volume/volume) Middlebrook albumin-dextrose-catalase (ADC) enrichment (Difco™, Becton, Dickinson Ltd., Oxford, UK), 0.05% Tween 80 (Sigma-Aldrich, Dublin, Ireland) and 0.40% (weight/volume) sodium pyruvate (Sigma-Aldrich Ltd., Dublin, Ireland) at 37°C. Bacterial cultures were grown to mid-logarithmic phase as determined by spectrophotometric analysis prior to the challenge experiments using the purified bovine MDM. Colony-forming unit (cfu) counting was performed using Middlebrook 7H11 medium (Difco™, Becton-Dickinson Ltd., Dublin, Ireland) containing 0.40% (volume/volume) sodium pyruvate (Sigma-Aldrich, Dublin, Ireland) and 10% (volume/volume) Middlebrook (ADC) enrichment (Difco™, Becton-Dickinson Ltd., Dublin, Ireland).

### *In vitro* challenge of bovine MDM with *M. bovis* and RNA extraction

**Figure 1:**
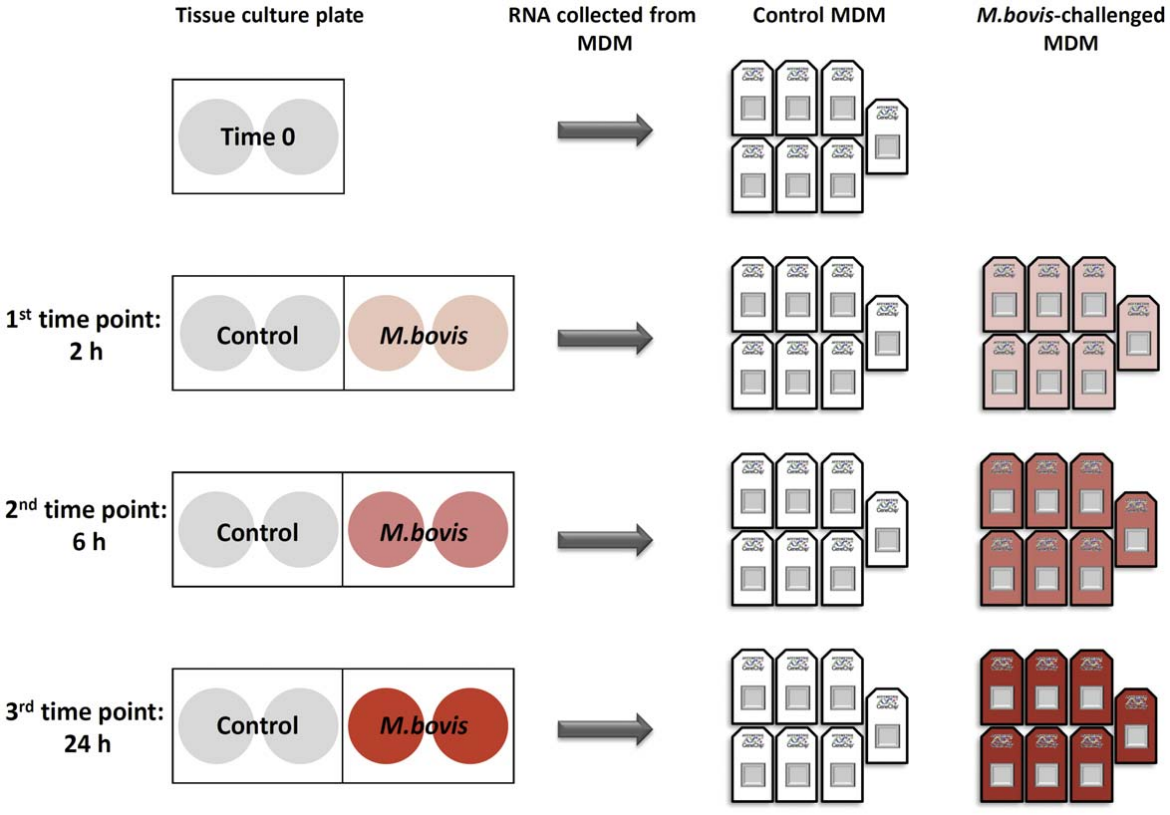
Schematic depicting the experimental design used in the current study. MDM were cultured in 24-well tissue culture plates (2×10^5^ cells per well) and challenged with *M. bovis* (MOI 2∶1). RNA was extracted from *M.bovis*-challenged and non-challenged control MDM at three time points post-challenge: 2 hours, 6 hours and 24 hours. In addition, RNA was extracted from a 0 hour non-challenged control to assess potential non-experimental changes in gene expression. The MDM lysates from replicate tissue culture wells (shaded) were pooled for RNA extraction. Global gene expression for the control and *M. bovis*-challenged MDM was analysed using the Affymetrix® GeneChip® Bovine Genome Array.

A schematic outlining the experimental design used in the current study is depicted in Figure 1. All *in vitro* challenge experiments including the preparation of non-challenge control MDM samples were performed in the CL3 laboratory. Prior to the *M. bovis* challenge experiments, MDM from two adjacent culture plate wells were lysed using RLT buffer from the RNeasy Mini kit (Qiagen Ltd., Crawley, UK) supplemented with 1% β-mercaptoethanol (Sigma-Aldrich Ireland Ltd., Dublin, Ireland) and pooled. These samples constituted the 0 hour control MDM.

MDM (seeded at 2×10^5^ cells per well) were challenged with *M. bovis* (4×10^5^ cells per well) prepared in antibiotic-free culture media (RPMI 1640 medium containing 15% heat inactivated FCS and 1% NEAA only). *M. bovis* cell counts were performed using a Petroff Hausser chamber (Fisher Scientific Ltd., Dublin, Ireland) following sterile filtering to prevent clumping using a 5 micron filter (Millipore Ireland Ltd., County Cork, Ireland). *M. bovis* cell numbers were then adjusted and 4×10^5^ cells were added to the appropriate wells giving an MOI of 2∶1. Subsequent cfu counting also yielded a mean MOI of 2∶1. Once challenged, MDM were then incubated at 37°C, 5% CO_2_ for 2 hours, 6 hours and 24 hours. For the non-challenged control samples at each time-point, antibiotic-free culture media (RPMI 1640 medium containing 15% heat inactivated FCS and 1% NEAA only) was added to each well. After 2 hours post-challenge, the media from all 6 hour and 24 hour challenge experiments was replaced with 0.5 ml fresh antibiotic-free culture media per well and re-incubated at 37°C, 5% CO_2_ until MDM were required for harvesting. Challenged and non-challenged control MDM were lysed and harvested using RLT-1% β-mercaptoethanol buffer (Qiagen Ltd., Crawley, UK) at the designated time points. For each treatment, MDM lysates from two culture plate wells were pooled and stored at −80°C until required for RNA extraction.

### RNA extraction and microarray analysis

All RNA extractions were performed in the CL3 laboratory using an RNeasy kit incorporating an on-column DNase treatment step (Qiagen Ltd., Crawley, UK) according to the manufacturer's instructions. RNA quantity and quality was assessed using a NanoDrop™ 1000 spectrophotometer (Thermo Fisher Scientific Inc., Waltham, MA, USA) and on an Agilent 2100 Bioanalyzer using an RNA 6000 Nano LabChip kit (Agilent Technologies Ltd., Cork, Ireland). All samples displayed a 260/280 ratio greater than 2.0 and RNA integrity numbers (RIN) greater than 8.5.

Total cellular RNA extracted from non-challenged control and *M. bovis*-challenged MDM from all seven animals at the 0 hour, 2 hour, 6 hour and 24 hour time points (giving a total of 49 samples) was prepared for global gene expression analyses using the pan-genomic high-density Affymetrix® GeneChip® Bovine Genome Array (Affymetrix UK Ltd., High Wycombe, UK; http://www.affymetrix.com). This array contains 24,072 probe sets representing over 23,000 gene transcript and includes approximately 19,000 UniGene clusters. cDNA labelling, hybridisation and scanning for the microarray experiments were performed by Almac Diagnostics Ltd. (Craigavon, Co. Armagh, Northern Ireland) using a one-cycle amplification/labelling protocol.

### Statistical analysis of microarray data

Affymetrix® GeneChip® Bovine Genome Array data were analysed using the BioConductor software project [18] (http://www.bioconductor.org) contained within the R statistical environment (http://www.r-project.org). Quality control analysis was performed on all 49 microarrays using the Simpleaffy software package [19] contained within BioConductor. Normalisation of raw data was performed using the Factor Analysis for Robust Microarray Summarization (FARMS) algorithm. The FARMS algorithm uses only perfect match probes and a quantile normalization procedure, providing both *P*-values and signal intensities [20]. Normalised data were then further subjected to filtering for informative probe sets using the Informative/Non-informative calls (I/NI-calls) software package within R [21]. I/NI-calls uses multiple probes for the same gene as repeated measures to quantify the signal-to-noise ratio of that specific probe set. Principal component analysis (PCA) was performed using data from all informative probe sets following all data filtering steps from the *M. bovis*-challenged and non-challenged control samples at the 2, 6 and 24 hour time points. The MultiExperiment Viewer version 4.7 software package [22] was used for PCA with Euclidean distance as the distance metric.

Differentially expressed genes were identified using the Linear Models for Microarray Data (LIMMA) package [23] contained within R. Genes displaying differential expression patterns between non-challenged control and M. *bovis*-challenged MDM at the 2, 6 and 24 hour time points were annotated using the Affymetrix® bovine gene annotation (http://www.affymetrix.com). The Benjamini-Hochberg multiple testing correction method [24] was applied to all differentially expressed genes to minimise the false discovery rate (FDR) and adjusted *P*-values for differentially expressed genes were calculated (adjusted *P*-value threshold 0.05).

Geometric mean fold-changes in gene expression are presented in the current study following back-transformation of mean log_2_ fold-changes obtained from statistical analysis of microarray data. For replicate probes for single genes, the average log_2_ expression fold-change was used and subsequently back-transformed. The negative reciprocals of the geometric fold-changes were calculated and are presented for downregulated genes. All microarray data were MIAME compliant [25] and have been submitted to the NCBI Gene Expression Omnibus (GEO) database [26] with experiment series accession number GSE33309.

### Systems biology analyses

Ingenuity® Systems Pathway Analysis (IPA; Ingenuity Systems, Redwood City, CA, USA; http://www.ingenuity.com) was used to identify canonical pathways and functional processes of biological importance within the list of differentially expressed genes. The Ingenuity® Knowledge Base contains the largest database of manually-curated and experimentally-validated physical, transcriptional and enzymatic molecular interactions. Furthermore, each interaction in the Ingenuity® Knowledge Base is supported by previously published information.

For IPA analysis, the Affymetrix® GeneChip® Bovine Genome Array was used as a reference gene set. All differentially expressed genes with an adjusted *P* value≤0.05 were included in the uploaded gene list and only differentially expressed genes mapping to molecules in the Ingenuity® Knowledge Base were used for systems analysis. Functional analysis of genes was performed using IPA to characterise the biological functions of the differentially expressed genes between the control and *M. bovis*-challenged MDM. For this, IPA performed an over-representation analysis that categorises the differentially expressed genes within the uploaded list into functional gene ontology (GO) categories. Each GO category in IPA is ranked based on the number of differentially expressed genes falling into each functional group. Right-tailed Fisher's exact tests were used to calculate a *P*-value for each of the biological functions assigned to the list of differentially expressed genes.

IPA contains a large library of known canonical pathways that were overlaid with the differentially expressed genes to identify major biological pathways associated with *M. bovis* infection in MDM. The significance of the association between differentially expressed genes and the canonical pathway was assessed using two methods: (1) a ratio of the number of molecules from the differentially expressed gene data set that map to the pathway, compared to the total number of molecules that map to the canonical pathway based on the reference gene list; and (2) a Fisher's exact test that generates a *P*-value for the assignment of the differentially expressed genes to a particular canonical pathway compared to the reference gene list. Canonical pathways were then overlaid with the expression values of the differentially expressed genes.

### Conventional cDNA preparation for real time quantitative reverse transcription PCR (qRT-PCR) analysis

cDNA was prepared from 80 ng of total RNA isolated from each sample analysed in the microarray study (including the 0 hour control RNA samples) using a High Capacity cDNA Reverse Transcription Kit (Applied Biosystems®, Life Technologies Corporation, Warrington, UK). cDNA conversions were performed in 20 µl reaction volumes using random primers as per the manufacturer's instructions. In addition, 80 ng of pooled RNA (comprising ∼10 RNA samples per pool) was included in non-reverse transcriptase (non-RT) control reactions to test for the presence of contaminating genomic DNA during real-time qRT-PCR analysis. All cDNA samples and non-RT controls were diluted 1∶8 (final concentration cDNA 0.5 ng/µl) using RNAse- and DNAse-free water and stored at −20°C prior to real time qRT-PCR analysis.

### Whole-transcriptome amplified cDNA preparation for real time quantitative reverse transcription PCR (qRT-PCR) analysis

To generate a repository of cDNA from each RNA sample, whole-transcriptome linearly amplified cDNA was prepared using the WT-Ovation™ RNA Amplification System (NuGEN Technologies Inc., Bemmel, the Netherlands). For this, 25 ng of template RNA was included in each amplification reaction (40 µl final volume) and all procedures were performed according to the manufacturer's instructions. In addition, non-template controls that included RNAse- and DNAse-free water instead of RNA template were also prepared using the WT-Ovation™ RNA Amplification System kit. Once amplification reactions were completed, the amplified cDNA and non-template controls were diluted 1∶1,000 using RNAse- and DNAse-free water and stored at −20°C prior to real time qRT-PCR analysis.

### Real time quantitative reverse transcription PCR (qRT-PCR) analysis and validation of microarray results

Intron-spanning primers were designed for each gene using the Primer3Plus package [27] and commercially synthesised (Eurofins MWG Operon, Ebersberg, Germany). Table S1 provides sequence information for all primer pairs used. Real time qRT-PCR reactions (20 µl final volume) were performed on 96-well plates using Fast SYBR® Green Master Mix (Applied Biosystems®, Life Technologies Corporation, Warrington, UK) on a 7500 Fast Real Time PCR System (Applied Biosystems®, Life Technologies Corporation, Warrington, UK) as per manufacturer's instructions. Real time qRT-PCR amplifications contained as template either: (a) 2 µl of the diluted conventionally-prepared cDNA/non-RT control (equivalent to 1.0 ng of total RNA); or (b) 2 µl of the diluted linearly amplified cDNA/non-RNA template control. A final concentration of 300 nM of each forward and reverse primer was included in each amplification reaction. Non-template real time qRT-PCR controls and a seven-point standard curve prepared from 1∶2 serial dilutions of pooled conventionally-prepared cDNA or linearly amplified cDNA from each *M. bovis*-challenged MDM sample were included on every real time qRT-PCR plate.

PCR thermal cycling conditions for each amplicon comprised one cycle at 50°C for 2 minutes, one cycle at 95°C for 20 seconds, followed by 40 cycles at 95°C for 3 seconds and 60°C for 30 seconds. A dissociation step was included for all amplifications to confirm the presence of single discrete PCR products of the expected size; this was further confirmed by visualisation of the amplification products on 2% agarose gels stained with 0.5 µg/ml ethidium bromide (Invitrogen™, Life Technologies Corporation, Paisley, UK).

### Statistical analysis of real time qRT-PCR data

All real time qRT-PCR data, including primer efficiency estimations using the standard curves, were analysed using the qbase^PLUS^ software package [28] (Biogazelle NV, Zwijnaarde, Belgium). Real time qRT-PCR efficiency correction and normalisation was performed using the peptidylprolyl isomerase A (cyclophilin A) gene (*PPIA*) as a reference gene based on GeNorm analysis performed by us in a previous study involving bovine MDM [17]. *PPIA* expression profiles were generated for both the conventionally-prepared and linearly amplified cDNA templates for all samples to ensure appropriate real time qRT-PCR efficiency correction and normalisation analyses were performed for all genes-of-interest.

Calibrated normalised relative quantities (CNRQ) of gene expression for each analysed sample, as generated by the qbase^PLUS^ package, were used to calculate fold-changes in expression for each gene. For this, the CNRQ value generated for each *M. bovis*-challenged MDM sample was divided by the CNRQ value generated for the non-challenged control MDM sample at the corresponding post-challenge time point from the same animal. Log_2_ fold-changes in gene expression were then used for the statistical analysis of all genes analysed using real time qRT-PCR. All statistical analyses were performed using the SPSS statistical package (IBM Corporation, Armonk, NY, USA) and the Minitab version 16 statistical package (Minitab Ltd., Coventry, UK).

Anderson-Darling tests of normality were applied to the residuals of the log_2_ fold-change values at each time point prior to statistical analysis to ensure the data conformed to a normal distribution—no significant departures from normality were observed for any of the genes analysed (*P*≥0.05). Two-tailed paired Student's *t*-tests were used to assess significant differences in mean log_2_ fold-changes in gene expression between the *M. bovis*-challenged MDM and the non-challenged control MDM at each time point post-challenge. Geometric mean fold-changes in expression were generated for each gene by back-transformation of the log_2_ fold-change data. The negative reciprocals of the geometric fold-changes were calculated and are presented for downregulated genes.

To validate the performance of the linearly amplified cDNA in real time qRT-PCR amplifications, the expression profiles of four genes (*CCL5*, *CCL20*, *IL1B* and *IL6*) were generated using both conventionally-prepared cDNA and linearly amplified cDNA. The log_2_ fold-changes in gene expression in the *M. bovis*-challenged MDM (relative to the control MDM) were then compared for both template types using regression analysis.

## Results

### A summary of differential gene expression at each time point post-challenge with *M. bovis*

**Figure 2:**
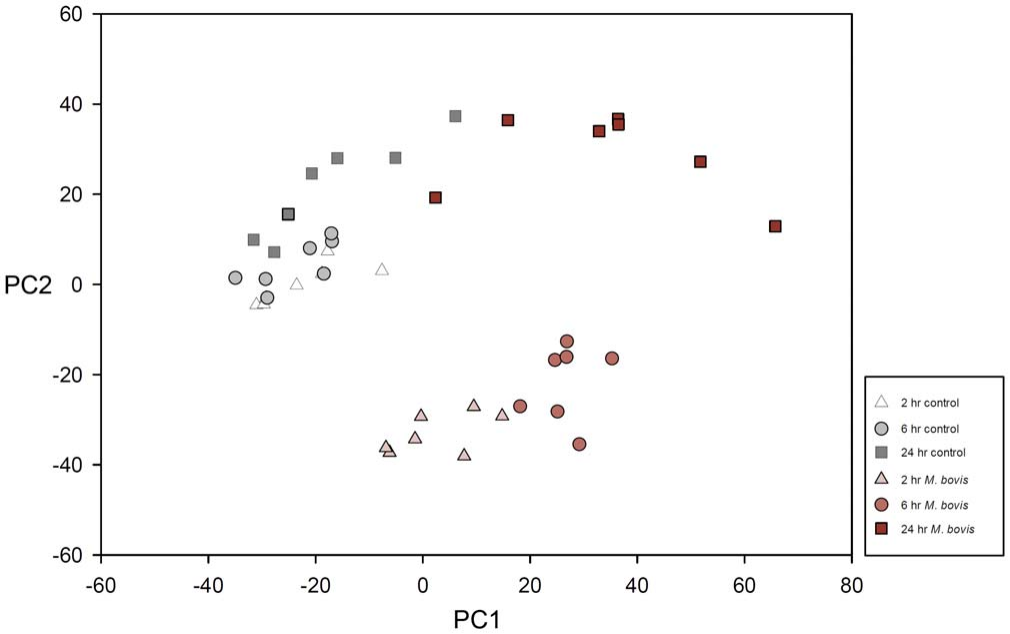
Principal component analysis for all individual control and *M. bovis*-challenged MDM at the 2 hour, 6 hour and 24 hour time points. Principal component (PC) 1 and PC2 are shown (accounting for 23.31% and 17.05% of the total variation, respectively). PCA was performed using data for all genes whose probes passed the data filtering process with Euclidean distance as the distance metric.

In total, 12,339 probe sets on the microarray passed filtering for informative probe sets using the I/NI-calls R package. PCA was performed using all informative transcripts that passed the data filtering process for all samples at the 2, 6 and 24 hour time points (Figure 2). Principal component 1 (PC1) primarily separates the challenge status (*i.e. M. bovis*-challenged and non-challenged controls) of the MDM samples, while PC2 delineates the *M. bovis*-challenged MDM with respect to time post-challenge. PC1 and PC2 account for 23.31% and 17.05% of the total variation in gene expression, respectively.

**Figure 3:**
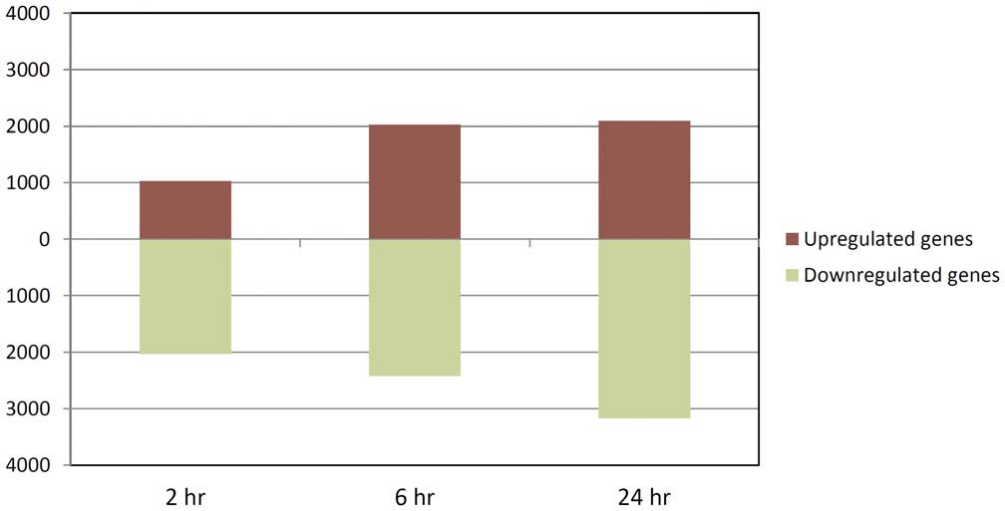
The number of significantly differentially expressed genes at each time point post-*M. bovis* challenge. The number of upregulated and downregulated genes in the *M. bovis*-challenged MDM relative to the control MDM at each time point are shown (adjusted *P*-value 0.05).

The gene expression data were then used to compare the gene expression profiles between the *M. bovis*-challenged MDM and non-challenged MDM samples across all time points, the results of which are summarised in Figure 3. This shows that the number of differentially expressed genes increased with each time point post-challenge, with the highest number of differentially expressed genes identified at the 24 hour time point. Furthermore, the number of downregulated genes exceeded the number of upregulated genes displaying increased gene expression in the challenged MDM at each time point.

### Analysis of global gene expression after 2 hours post-challenge with *M. bovis*

At 2 hours post-challenge, a total of 3,529 transcripts, representing 3,064 unique genes, were identified as being differentially expressed between the *M. bovis*-challenged and non-challenged MDM (adjusted *P*-value≤0.05). Of these, 1,253 transcripts (representing 1,031 genes) were upregulated while the remaining 2,276 transcripts (representing 2,033 genes) were downregulated in the *M. bovis*-challenged MDM. Notably, the relative fold-change in expression was markedly higher for the upregulated genes compared to the downregulated genes at this time point. A complete list of the differentially expressed genes and the relative fold-change in expression at the 2 hour time point post-challenge are provided in Table S2.

Further inspection of the differentially expressed genes revealed that many of the highly upregulated genes at this time point had immune-related functions. These included NF-κB-inducible chemokine and cytokine genes, such as the (C-C motif) ligand 20 gene (*CCL20*); the interleukin 1, beta gene (*IL1B*); the chemokine (C-X-C motif) ligand 2 gene (*CXCL2*); the interleukin 1, alpha gene (*IL1A*); the tumour necrosis factor gene (*TNF*) and the interleukin 6 gene (*IL6*).

**Figure 4:**
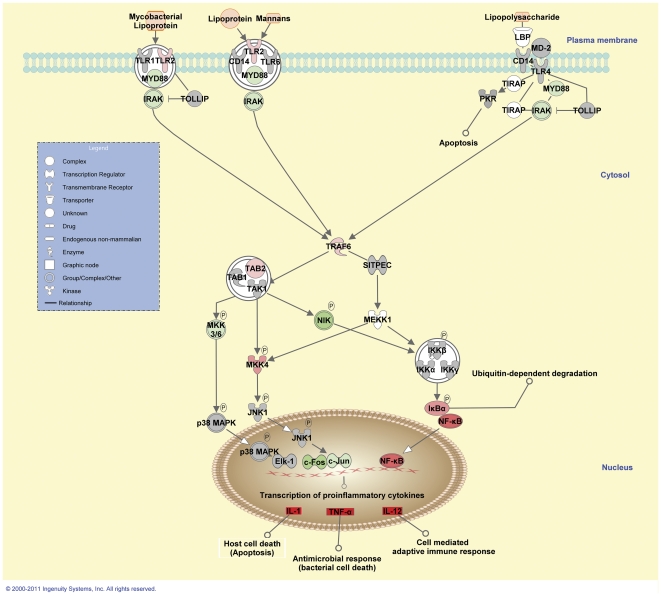
Differential gene expression in the TLR signalling pathway 2 hours post-*M. bovis* challenge. Genes within the TLR signalling pathway showing differential expression are highlighted in colour. Colour intensity indicates the degree of upregulation (red) or downregulation relative to the control MDM. Grey shading indicates genes that were not differentially expressed; white shading represents genes in the pathway not represented on the microarray; mycobacterial PAMPs are shaded orange.

Several genes encoding proteins associated with TLR signalling were also differentially expressed 2 hours post-*M. bovis* challenge. These included *TLR2* (upregulated); the myeloid differentiation primary response (88) gene (*MYD88*) [downregulated]; the interleukin-1 receptor-associated kinase 1 gene (*IRAK1*) [downregulated]; the interleukin-1 receptor-associated kinase 4 gene (*IRAK4*) [downregulated]; the toll-like receptor adaptor molecule 1 gene (*TICAM1*) [upregulated; note: this gene was not present on the microarray but was analysed using real time qRT-PCR]; the TNF receptor-associated factor 6 gene (*TRAF6*) [upregulated]; the TNF receptor-associated factor 3 gene (*TRAF3*) [upregulated]; the TGF-beta activated kinase 1/MAP3K7 binding protein 2 gene (*TAB2*) [upregulated] and the nuclear factor of kappa light polypeptide gene enhancer in B-cells inhibitor, alpha gene (*NFKBIA*) [upregulated]. The genes encoding NF-κB1 (*NFKB1*) and NF-κB2 (*NFKB2*) were also upregulated in the *M. bovis*-challenged MDM at this time point (Figure 4). *TLR4* and the gene encoding IFN-γ (*IFNG*)―two genes previously reported to play an important role in the immune response to mycobacterial infection [4]―were not differentially expressed at the 2 hour time point.

Of the 3,064 differentially expressed genes identified at 2 hours post-challenge, 1,916 mapped to unique molecules represented in the Ingenuity® Knowledge Base. Functional categorisation analysis using IPA showed that the differentially expressed genes had roles involved in the regulation of gene transcription, the immune response and cell signalling (Table S3). IPA was also used to identify the top ranking canonical pathways enriched for differentially expressed genes in response to *M. bovis* challenge. Many of the top ten ranking canonical pathways identified had an immunological role and included *Interleukin 10 signalling* (1^st^ ranked pathway), *B cell activating factor signalling* (2^nd^ ranked pathway), *CD40 signalling* (4^th^ ranked pathway) and *IL6 signalling* (5^th^ ranked pathway) (Table S4).

### Analysis of global gene expression after 6 hours post-challenge with *M. bovis*

Comparison of the *M. bovis*-challenged MDM and non-challenged control MDM 6 hour post-challenge, revealed 5,211 differentially expressed transcripts, comprising 4,451 differentially expressed genes. Of these, 2,458 transcripts (representing 2,027 unique genes) were upregulated and 2,753 transcripts (representing 2,424 unique genes) were downregulated in the *M. bovis*-challenged MDM (Table S5). As with the 2 hour time point, the relative fold-change in expression was markedly higher for the upregulated genes compared to the downregulated genes after 6 hours post-challenge.

Immune-related genes were among the differentially expressed genes showing the highest relative increase in expression. The identity of the genes displaying the highest fold-change in expression at the 6 hour time point were similar to those detected at the 2 hour time point, among which were *CCL20*, *IL1B*, *TNF* and *IL6*. *IFNG* was also upregulated in the *M. bovis*-challenged MDM at this time point. In addition, a small number of genes encoding proteins belonging to TLR signalling pathways displayed differential expression in the *M. bovis*-challenged MDM, included *TLR3* (upregulated), *IRAK1* (downregulated), *IRAK4* (downregulated), *TICAM2* (upregulated) and *TRAF3* (upregulated). The genes encoding TLR2, TLR4, MyD88, TICAM1 and TRAF6 were not differentially expressed at this time point.

Of the 4,451 differentially expressed genes identified at 6 hours post-challenge, 2,850 mapped to unique molecules represented in the Ingenuity® Knowledge Base. Functional analysis comparing *M. bovis*-challenged and non-challenged MDM transcriptomes revealed an enrichment of genes with immunological, apoptotic and cellular signalling functions (Table S6). Cellular pathways associated with the immune response were also among the top ranking canonical pathways identified by IPA, several of which were similar to those identified at the 2 hour time point. These included *IL6 signalling* (5^th^ ranked pathway), *CD40 signalling* (8^th^ ranked pathway) and *IL10 signalling* (11^th^ ranked pathway).

**Figure 5:**
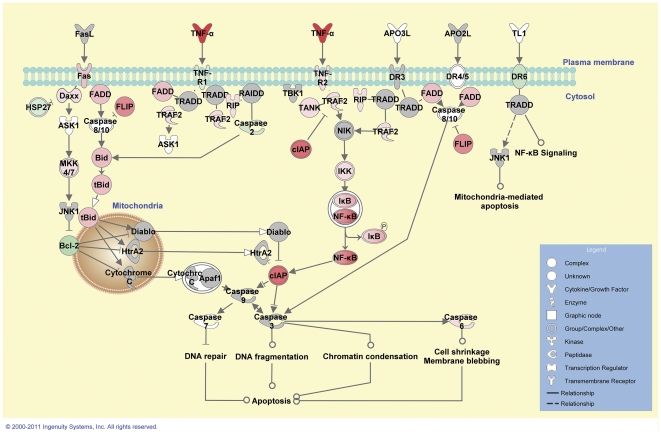
Differential gene expression associated with apoptosis 6 hours post-*M. bovis* challenge. Genes associated with apoptosis showing differential expression are highlighted in colour. Colour intensity indicates the degree of upregulation (red) or downregulation relative to the control MDM. Grey shading indicates genes that were not differentially expressed; white shading represents genes in the pathway not represented on the microarray.

*Death receptor signalling* was identified as the fourth highest ranking canonical pathway 6 hours post *M. bovis*-challenge. Given the previously identified role of programmed cell death during mycobacterial infection *in vitro* [14], [15], the pattern of differential gene expression in this pathway is depicted in Figure 5. Interestingly, the *Role of retinoic-acid-inducible I* (RIGI)*-like receptors in anti-viral innate immunity* (14^th^ ranked pathway), a cellular pathway primarily involved in the host recognition of viral PAMPs was also listed among the top ranking canonical pathways after 6 hours post-challenge with *M. bovis* (Table S7).

### Analysis of global gene expression changes 24 hours post-challenge with *M. bovis*

6,150 differentially expressed transcripts representing 5,267 unique genes were identified through comparison of the global gene expression profiles of the *M. bovis*-challenged MDM and the non-challenged control MDM 24 hours post-challenge (Table S8). Of these, 2,537 transcripts (comprising 2,093 unique genes) were upregulated and 3,613 transcripts (comprising 3,174 unique genes) were downregulated in the *M. bovis*-challenged MDM. Among the genes with immunological functions displaying high levels of increased relative gene expression in the *M. bovis*-challenged MDM included the chemokine (C-C motif) ligand 8 gene (*CCL8*), the chemokine (C-X-C motif) ligand 11 gene (*CXCL11*), *IL1B* and *IL6*. *TNF* and *IFNG* also displayed increased relative expression in the *M. bovis*-challenged MDM at this time point.

Of the 5,267 differentially expressed genes identified at 24 hours post-challenge, 3,377 mapped to unique molecules represented in the Ingenuity® Knowledge Base. As with the 2 hour and 6 hour time points, functional categorisation of the differentially expressed genes 24 hours post-challenge revealed an enrichment of genes involved in the host immune response, particularly the activation and development of the adaptive immune response (Table S9). Furthermore, many of the top ranking canonical cellular pathways identified 24 hours post-*M. bovis* challenge were associated with adaptive immune function. These included *Communication between innate and adaptive immune cells* (3^rd^ ranked pathway); *Interferon signalling* (8^th^ ranked pathway); *T helper cell differentiation* (9^th^ ranked pathway) and *IL6 signalling* (12^th^ ranked pathway) (Table S10).

**Figure 6:**
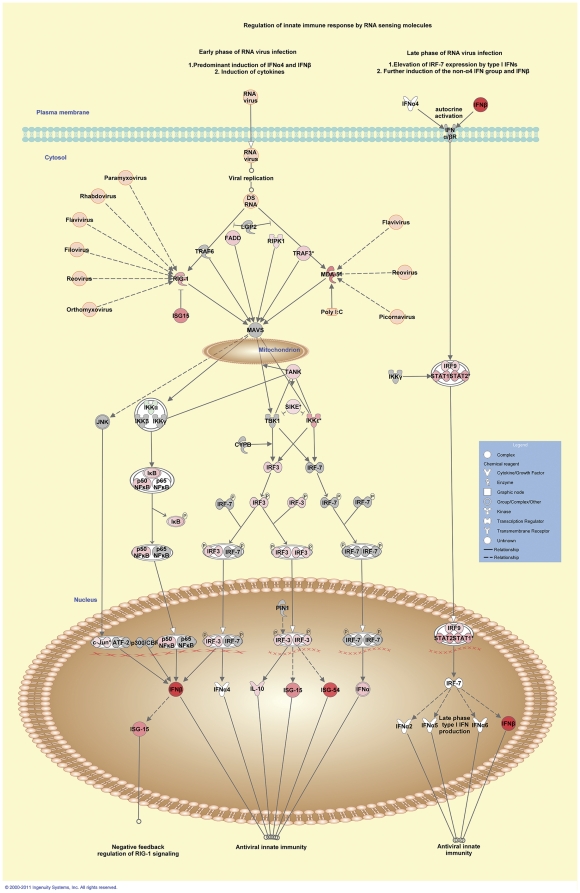
Differential gene expression associated with intracellular pathogen recognition receptors 24 hours post-*M. bovis* challenge. Genes associated with intracellular PRR signalling showing differential expression are highlighted in colour. Colour intensity indicates the degree of upregulation (red) or downregulation relative to the control MDM. Grey shading indicates genes that were not differentially expressed; white shading represents genes in the pathway not represented on the microarray; viral PAMPs are shaded orange.

As with the 6 hour post-challenge results, systems analysis also identified intracellular PRRs as playing a role in *M. bovis* infection *in vitro*. This was evidenced by the identification of *Activation of interferon regulatory factors by cytosolic pattern recognition receptors* (5^th^ ranked pathway, presented in Figure 6) and the *Role of RIG-I-like receptors in antiviral innate immunity* (7^th^ ranked pathway) among the top ranking canonical pathways at the 24 hour time point.

### Real time quantitative reverse transcription PCR (qRT-PCR) analysis and validation of microarray results

A panel of the 16 top ranking differentially expressed genes identified through analysis of the microarray data were selected for real time qRT-PCR analysis and validation. Selection of these genes was based largely on the highest fold-change in increased and reduced relative expression and the lowest adjusted *P*-value across all three time points. These genes were: *AREGB, CCL4*, *CCL5*, *CCL20*, *CD40*, *CFB*, *CXCL2*, *FOS, IL1B*, *IL6*, *IL15*, *IRF1*, *NFKB2*, *PIK3IP1*, *SPRY2* and *TNF*. In addition, the expression profile of *TICAM1*, which was not represented on the Affymetrix® GeneChip® Bovine Genome Array, was analysed using real time qRT-PCR.

Of the total of 17 genes analysed using real time qRT-PCR, eight genes (*CCL4*, *CD40*, *CFB*, *CXCL2*, *IL15*, *IRF1*, *NFKB2* and *TNF*) were analysed using conventionally-prepared cDNA template only; five genes (*AREGB*, *FOS*, *PIK3IP1*, *SPRY* and *TICAM1*) were analysed using the linearly amplified cDNA template only; while the remaining four genes (*CCL5*, *CCL20*, *IL1B* and *IL6*) were analysed using both the conventionally-prepared cDNA and the linearly amplified cDNA templates in order to validate the performance of the linearly amplified cDNA in real time qRT-PCR analyses.

To validate the performance of the linearly amplified cDNA in real time qRT-PCR amplifications, the expression profiles generated for the *CCL5*, *CCL20*, *IL1B* and *IL6* genes using conventionally-prepared and linearly amplified cDNA were compared. For this, regression analysis was performed using the log_2_ fold-changes in relative gene expression for the *M. bovis*-challenged MDM analysed for the two different cDNA templates across all three post-challenge time points, revealing a high correlation for all four genes (*r^2^*≥0.65, *P*≤0.001) (Figure S1). Furthermore, the geometric mean fold-changes in expression and Student's *t*-test statistics in the *M. bovis*-challenged MDM relative to the control MDM for these genes were similar across all three time points (Figure S2 and Table S11). These results suggest that no preferential amplification bias of gene transcripts or amplification artefacts occurred during the preparation of the linearly amplified cDNA. Consequently, we concluded that the use of the linearly amplified cDNA was suitable in the current study.

**Figure 7:**
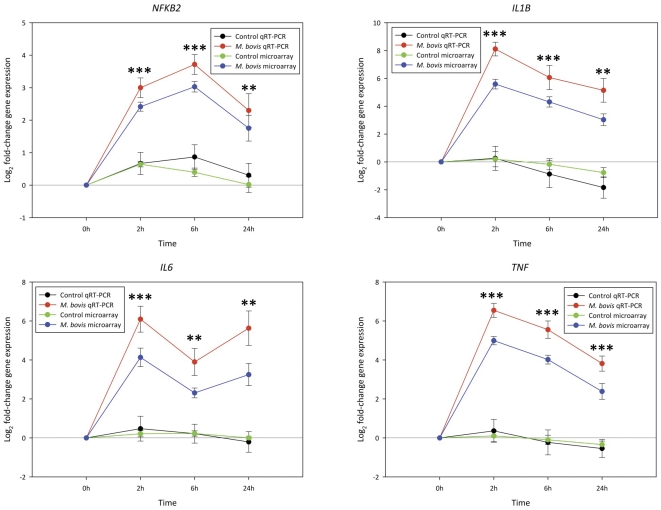
Real time qRT-PCR analysis of the *IL1B*, *IL6*, *NFKB2* and *TNF* genes following *M. bovis* challenge. Log_2_ fold-changes in expression in the *M. bovis*-challenged MDM relative to the non-challenged control MDM at all three time points are shown. For comparison, the expression profiles for these genes based on the microarray data are also shown. The significance of the fold-changes in expression for each gene based on the real time qRT-PCR analysis only are denoted by asterisks in the figure (*P≤0.05, **P≤0.01, ***P≤0.001). The fold-changes calculated for each gene from the microarray data in the *M. bovis*-challenged MDM for each gene at each time point were significant (adjusted *P*-value ≤ 0.05). In addition, the log_2_ fold-change in expression for the control MDM at each time point relative to the 0 hour control MDM are also shown for both the microarray and real time qRT-PCR data; no significant differences in gene expression between the control MDM relative to the 0 hour control was observed for these genes at each time point (*P*≥0.05).

The relative fold-changes in expression between the *M. bovis*-challenged and control MDM for the *IL1B*, *IL6*, *NFKB2* and *TNF* genes as determined through real time qRT-PCR analysis (using the conventionally-prepared cDNA as template) are depicted graphically in Figure 7, while fold-changes in expression for all other genes analysed are shown graphically in Figure S3. In addition, comparisons between the results from the real time qRT-PCR and microarray analyses for all genes are given in Table S12. These results showed that twelve genes (*CCL4*, *CCL5*, *CCL20*, *CD40*, *CFB*, *CXCL2*, *IL1B*, *IL6*, *IL15*, *IRF1*, *NFKB2* and *TNF*) were significantly upregulated across all three time points (*P*≤0.01). Two genes (*AREGB* and *FOS*) displayed significant downregulation in the *M. bovis*-challenged MDM samples across all time points (*P*≤0.05). *TICAM1* was differentially expressed (upregulated) only at the 2 hour time point (*P*≤0.001). *PIK3IP1* showed significant decreased relative expression in the *M. bovis*-challenged samples at the 2 hour and 6 hour time points (*P*≤0.05); however, this gene was not differentially expressed at the 24 hour time point. *SPRY2* displayed significant (*P*≤0.05) decreased relative expression in the *M. bovis*-challenged MDM at the 2 hour time point but was not differentially expressed after 6 hours and 24 hours post-challenge. Comparison of the real time qRT-PCR expression profiles of each gene for the 2 hour, 6 hour and 24 hour non-challenged control MDM relative to the 0 hour non-challenged control MDM did not reveal significant differential expression (*P*≥0.05) for 16 of the 17 analysed genes; only the expression profile of the *AREGB* gene in the 0 hour control MDM differed from the control MDM at 2 hours and 6 hours (*P*≤0.01).

Gene expression profiles for all 16 genes analysed using both real time qRT-PCR and microarray platforms were largely the same across all three time points. Only the expression profile of the *SPRY* gene at 6 hours and 24 hours post-challenge and the *PIK3IP1* gene at 24 hours post-challenge did not display significant differential expression (*P*≥0.05) between the *M. bovis*-challenged and non-challenged control MDM based on real time qRT-PCR analysis but displayed significant reduced relative expression (adjusted *P*-value threshold ≤0.05) based on the microarray results at these time points. The observed discrepancies between the microarray and real time qRT-PCR data for the *SPRY2* and *PIK3IP1* genes at the 6 hour and 24 hour time points may reflect differences in the sensitivity of the two analytical methods used and/or differences in the mRNA transcripts targeted by the probes (microarray) and primer pairs (real time qRT-PCR) used for the two forms of gene expression analysis [29], [30].

## Discussion

The host immune response to mycobacterial infection is highly complex and involves interaction between innate and adaptive immune mechanisms. The macrophage is among the first innate immune cell types to encounter mycobacteria and the response elicited by these cells is key to the control of infection [3], [31]. Consequently, investigation of gene transcriptional changes in macrophages following mycobacterial challenge *in vitro* provides a model for understanding the complex host-pathogen interactions and innate cellular signalling pathways that underlie the host immune response to mycobacterial infection. Therefore, for the current study, we have investigated gene expression in bovine MDM in response to *M. bovis* challenge *in vitro* using the pan-genomic high-density Affymetrix® GeneChip® Bovine Genome Array at 2, 6 and 24 hours post-challenge. The gene expression data from these analyses were used to investigate and reconstruct key host regulatory networks involved during early mycobacterial infection.

It is important to note that MDM are not the primary cell type that recognise *M. bovis* during the course of natural infection and that bovine alveolar macrophages would be a more appropriate cell type to analyse the host transcriptional response to *M. bovis* challenge *in vitro*. However, MDM are widely used for *in vitro* challenge experiments involving mycobacteria as they best mimic the *in situ* status of infection compared to other cell types used for experimental infection (*e.g.* cell lines), particularly in experiments where alveolar macrophages are difficult to source [32]–[38]. Furthermore, bovine MDM are easily prepared from peripheral blood mononuclear cells using standard procedures, whereas the preparation of bovine alveolar macrophages requires *post-mortem* lung lavages [14], [39]. Finally, blood macrophages do recognise mycobacteria during secondary phases of infection that occur when bacilli disseminate from infected primary alveolar macrophages [40]; therefore, the results presented in the current study may also provide insight into the macrophage immune mechanisms that occur during the progression of infection.

It should also be noted that the culturing of *M. bovis* as performed in this study involved the use of growth media containing Tween 80, a non-ionic surface-active detergent that affects the mycobacterial cell wall composition, causing a reduction in virulence [41]. However, Tween 80 is routinely added to liquid media to reduce cell clumping (which can affect colony-forming unit [cfu] counts), therefore enabling homogeneous cell suspensions of mycobacteria to be obtained for *in vitro* macrophage challenge experiments [41], [42]. As such, the current study is directly comparable to recent studies investigating the interaction of *M. tuberculosis* complex bacilli with host macrophages [14], [32], [33], [43]–[45].

### The patterns of global gene expression support the association of *M. bovis* infection with repression of host gene expression

Comparison of the gene expression profiles in the *M. bovis*-challenged MDM relative to the control MDM at each time point revealed an increase in the number of differentially expressed genes across the three post-challenge time points analysed. Notably, the number of downregulated genes exceeded the number of upregulated genes at each time point; however, the fold-change in expression for the upregulated genes was much greater than that for the downregulated genes at each time point. These findings support previous work by us and others showing that mycobacterial infection *in vitro* and *in vivo* is associated with pan-genomic and immuno-specific gene repression [46]–[49]. However, at each time point post-challenge, the mean fold-change in expression for upregulated genes was markedly higher than the mean fold-change in expression for downregulated genes. Furthermore, many of the upregulated genes displaying the highest fold-increase in expression at each time point post-challenge had a known immunological role, while many of the genes displaying the greatest reduction in expression had no obvious immune function―a finding that highlights the complex nature of the interaction between the macrophage and *M. bovis* during infection.

### Analysis of global gene expression signatures 2 hours post-challenge with *M. bovis* support a role for MyD88-independent signalling

Host innate immune recognition of mycobacterial pathogens is largely regulated through the engagement of macrophage PRRs with mycobacterial PAMPs. TLRs are among the most important PRRs for the recognition of mycobacterial PAMPs during the early stages of infection, particularly the cell surface TLR2 (acting alone or as a heterodimeric complex with TLR1 or TLR6) and TLR4 receptors [4], [50]. More recently, membrane-associated cytosolic TLRs, such as TLR8 and TLR9, and the cell surface TLR5 receptor have also been shown to play a role in host detection of mycobacteria [51]-[53].

TLR2 and TLR4 signalling is predominantly mediated through the adaptor protein, MyD88, the activation of which results in the initiation of an inflammatory signalling cascade involving IRAK1, IRAK4 and TRAF6. Activated TRAF6 can interact with the regulatory components of the nuclear receptor subfamily 2, group C, member 2 (NR2C2) complex (also called the TGF-b activated kinase [TAK1] complex), culminating in the activation of TAK1, which can phosphorylate downstream targets, such as the IκB kinases (IKKs). Phosphorylation of the IKK complex catalyses the degradation of the NF-κB inhibitors (IKBs) resulting in the release of the NF-κB complex, which translocates to the nucleus and directs the transcription of many immune-related genes, including inflammatory cytokines and chemokines necessary to prime the adaptive immune response [54], [55].

A MyD88-independent pathway (also called the TRIF-dependent pathway) has also been reported as an alternative signalling route for TLR4 and as the primary route for the membrane-associated cytosolic TLR3 receptor. MyD88-independent signalling involves the recruitment of at least two adaptor proteins: TICAM1 (also called TRIF) and TICAM2 (also called TRAM). TICAM1 can activate TRAF3 to induce interferon regulatory factor 3 (IRF3)-mediated transcription of type I interferons (IFN), or it can form heterodimeric signalling complexes with the TNFRSF1A-associated via death domain (TRADD) and ralA binding protein 1 (RALBP1, also called RIP1) proteins that induce both NF-κB- and mitogen-activated protein kinases (MAPK)-mediated transcription of inflammatory cytokines [54], [55].

In the current study, inspection of the differentially expressed genes at the 2 hour time point confirm a role for TLR-mediated recognition of *M. bovis* in the challenged MDM. The only differentially expressed TLR gene at this time point was *TLR2* (upregulated), while many of the genes encoding proteins involved in TLR signalling were also shown to be differentially expressed in the *M. bovis*-challenged MDM at this time point. These included *MYD88* (downregulated); *IRAK4* (downregulated); *IRAK1* (downregulated); *TICAM1* (upregulated); *TRAF6* (upregulated); *TRAF3* (upregulated); *TAB2* (upregulated) and *NFKBIA* (upregulated). *TAB3* and *NR2C2* were not differentially expressed.

The induction of *TLR2* 2 hours post-challenge suggest that this PRR is involved in the early macrophage recognition of *M. bovis*. However, the suppression of the genes encoding the MyD88 adaptor and the IRAK4 and IRAK1 kinases at this time point post-challenge suggests that MyD88-dependent signalling is repressed during *M. bovis* infection. Alternatively, these results may point to the activation of a macrophage TLR2-dependent/MyD88-independent signalling pathway involving alternative adaptor protein(s), such as TICAM1, during *M. bovis*-challenge *in vitro*. Furthermore, the increased relative expression of *TRAF6* and *TAB2* 2 hours post-infection suggests that this signalling cascade bypasses TRADD (the gene encoding TRADD was downregulated) and RALBP1 (the gene encoding RALBP1 was downregulated) and is relayed directly to the IKKs, inducing NF-κB-directed transcription. Although there is little published work on TLR2-dependent/MyD88-independent pathway(s), previous research involving *in vitro* infections of murine peritoneal macrophages with heat-killed *M. tuberculosis* was shown to result in the expression of the secretory leukocyte peptidase inhibitor gene (*SLPI*) via a TLR2-dependent/MyD88-independent pathway [56]. Similarly, microarray-based analysis of the murine bone marrow-derived macrophage transcriptome following infection with virulent *M. tuberculosis in vitro* was also shown to induce MyD88-independent signalling pathways, possibly involving TLR2 [57].

The repression of MyD88-dependent signalling may represent a transcriptional signature of *M. bovis*-mediated subversion of host immune responses. Indeed, inhibition of IFN-γ-induced MHC class II expression in *M. tuberculosis*-infected macrophages has been shown to involve MyD88-dependent signalling mediated through TLR2 [58], [59]. Thus, the suppression of TLR2-MyD88-dependent signalling may represent one mechanism for immuno-evasion by the mycobacterial pathogen enabling persistence within the host macrophage during infection. In contrast, the activation of MyD88-independent signalling may present an alternative route used by host macrophages to circumvent suppressed MyD88-dependent signalling and induce downstream transcription factors which promote chemokine and cytokine production during infection.

It is also important to note that as this is a transcriptomics study, the functional role of various pre-existing TLR adaptor proteins in macrophage cellular pathways during *M. bovis* infection cannot be assessed at the RNA level; consequently, their involvement in these pathways cannot be fully excluded. Further work involving both transcriptomic and proteomic platforms is required to investigate fully a role for macrophage TLR2-dependent/MyD88-independent signalling pathways in response to mycobacterial infection.

### A role for intracellular PRR-signalling in the control of *M. bovis* infection in vitro

Inspection of the list of differentially expressed genes demonstrated that the expression of several genes encoding intracellular PRRs―which are generally associated with the detection of viral PAMPs, such as viral RNA―is modulated during *M. bovis* challenge *in vitro*. This was further supported by systems analysis, which identified *Signalling by RIG-I-like receptors in anti-viral innate immunity* and *Activation of interferon regulatory factors by cytosolic pattern recognitions receptors* among the top ranking canonical pathways at 6 hours and 24 hours post-*M. bovis* challenge, respectively.

RIG-I-like receptors (RLRs) represent a group of RNA helicases that function as cytosolic sensors of PAMPs within viral RNA or processed self RNA ligands to elicit innate immune responses to control infection. To-date, three RLR protein members have been identified: retinoic acid-inducible gene I (RIG-I, encoded by *DDX58*); melanoma differentiation associated factor 5 (MDA5, encoded by *IFIH1*); and laboratory of genetics and physiology 2 and a homolog of mouse D11lgp2 (LPG2, encoded by *DHX58*) [60]. In general, intracellular signalling mediated by RLRs results in the activation of downstream transcription factors that culminates in the production of type I IFN and antiviral proteins that control viral infection [60]. Notably, all three genes were differentially expressed in the current study at the 6 hour and 24 hour time points: *DDX58* (downregulated at 6 hours and upregulated at 24 hours), *IFIH1* (upregulated at 6 hours and 24 hours) and *DHX58* (upregulated at 24 hours).

Genes encoding members of the TLR3 signalling pathway also displayed differential expression in the *M. bovis*-challenged MDM at the 6 hour and 24 hour time points. *TLR3* encodes a membrane-bound intracellular PRR that localises to endosomes and is involved in the recognition of viral PAMPs, such as double-stranded reoviral RNA [54]. TLR3-mediated signalling occurs through the TICAM1 adaptor which recruits TRAF3 to activate IRF3-mediated transcription of type I IFN. In the current study, *TLR3* was upregulated in the *M. bovis*-challenged MDM samples at 6 hours and 24 hours post-challenge, while upregulation of *TRAF3*, *IRF3* and *INFB1* was also observed at the 24 hour time point. *TICAM1* was not differentially expressed at either the 6 hour or 24 hour time points.

The involvement of RLRs and TLR3 in mediating the macrophage response to *M. bovis* infection is intriguing given their well-documented role in the detection of viral PAMPs. However, transfection experiments have shown that murine bone marrow-derived macrophage production of type I IFN in response to RNA isolated from *Legionella pneumophila* (a gram-negative bacterium that replicates in host macrophages and causes severe pneumonia) is mediated via RIG-I [61]; while RNA from *Helicobacter pylori* (a gram positive bacterium that causes gastritis) was also shown to induce type I IFN production via a RIG-I-mediated signalling pathway in mouse dendritic cells [62]. Moreover, increased *TLR3* RNA and protein expression has been reported in human leukocytes following *in vitro* stimulation with bacterial lipopolysaccharide (LPS), while increased relative *TLR3* expression has been reported by us in the transcriptome of peripheral blood leukocytes (PBL) from cattle displaying active BTB compared to the PBL transcriptome of non-infected control animals [48], [62].

Collectively, these findings support a role for cytosolic RLRs and TLR3 during host infection with intracellular bacterial pathogens. It has been proposed that activation of intracellular PRRs by bacterial PAMPs is due to the translocation of bacterial RNA into host cells, or is a consequence of the generation of host-derived RNA ligands caused by the pathogen-mediated disruption of host cellular pathways during infection [61]. Another possibility is that intracellular PRRs mediate autophagy in infected macrophages, which is increasingly regarded as a key anti-mycobacterial host defence mechanism [63]–[65]. Autophagy is a complex cellular process in which host cell cytosolic components, such as damaged or surplus organelles, are engulfed in double-membrane vesicles called autophagosomes and fused with late endosomes or lysosomes to degrade their contents. However, further work is required to investigate a role for RLR- and TLR3-mediated autophagy in response to mycobacterial infection.

### Pro- and anti-apoptotic global transcriptional signatures associated with *M. bovis* challenge in vitro

Systems analysis of the differentially expressed genes revealed significant enrichment for genes involved in programmed cell death (apoptosis), particularly at 6 hours post-*M. bovis* challenge. Apoptosis is a complex biological process triggered by the engagement of protein receptors and ligands that initiate a caspase-directed signalling cascade. In particular, caspase activation and subsequent apoptosis is elicited upon the binding of TNF-α and the Fas ligand protein (FASLG) to their respective cell surface receptors, TNF-R1 or TNF-R2 and the FAS (TNF receptor superfamily member 6) receptor. This interaction results in the initiation of a death-inducing signal complex that involves recruitment of FADD, caspase 8 (CASP8) and CASP10, triggering a signalling cascade that culminates in the activation of effector caspases (such as CASP3, CASP6 and CASP7) and other non-caspase enzymes that induce DNA fragmentation, mitochondrial membrane permeability and degradation, and apoptotic vesicle formation [66].

In the current study, genes encoding members of the caspase family of proteins together with several pro- and anti-apoptotic mediators were differentially expressed following challenge with *M. bovis*. *TNF* was among the pro-apoptotic genes displaying upregulated gene expression in the *M. bovis*-challenged MDM at the 6 hour time point; however, the gene encoding the pro-apoptotic TNF-R1 receptor (*TNFRSF1A*) was downregulated at this time point. In contrast, the gene encoding the pro-apoptotic FAS (TNF receptor superfamily member 6) receptor (*FAS*) was upregulated in these MDM samples.

*FADD* and the death-domain associated protein gene (*DAXX*), which encode adaptor proteins that mediate pro-apoptotic signals from activated TNF-R1 [67] and the FAS receptor [68], respectively, were also upregulated after 6 hours post-challenge. Induction of the BH3 interacting domain death agonist gene (*BID*) was also observed; the activated form of the BID protein, truncated BID (tBID), forms heterodimeric complexes with other pro-apoptotic proteins causing mitochondrial membrane permeability and the release of cytochrome C, which activates the effector caspases to induce apoptosis [66].

Several anti-apoptotic genes also exhibited differential expression at the 6 hour time point. These included the gene encoding TNF-R2 (*TNFRSF1B*) [upregulated] that recruits the encoded product of the apoptosis inhibitor baculoviral IAP repeat containing 3 gene (*BIRC3*; downregulated) [69]. Notably, expression of the B-cell CLL/lymphoma 2 gene (*BCL2*) that encodes a protein that inhibits mitochondrial membrane permeability and prevents apoptosis [70] was suppressed following *M. bovis* challenge *in vitro* at this time point.

Macrophage apoptosis is increasingly regarded as a host innate immune mechanism in controlling mycobacterial infection by containing and limiting mycobacterial growth [71]. Recently, microarray-based studies of mycobacterial infection of bovine macrophages *in vitro* revealed over-representation of genes associated with apoptosis among the list of differentially expressed genes [14], [15]. The detection of apoptotic signatures of gene expression here supports this earlier work and indicates that apoptosis is an early response of host MDM to *M. bovis* infection.

Although analysis of the differentially expressed genes following *M. bovis*-challenge largely supports induction of a pro-apoptotic response in these cells, presumably to eliminate the intracellular pathogen, the increased relative expression of several anti-apoptotic genes suggests that this process is highly regulated. Anti-apoptotic signals may represent a host mechanism to limit the amount of cell death following infection and may also enable improved antigen-presentation by infected macrophages to T cells, resulting in either elimination of the pathogen or enhanced granuloma formation. It is also possible that the pro- and anti-apoptotic signals detected here represent transcriptional signatures of “bystander” apoptosis, whereby uninfected macrophages undergo apoptosis following contact with mycobacteria-infected macrophages [15], [72].

Alternatively, the induction of anti-apoptotic genes may signify an immuno-subversion mechanism used by the pathogen that postpones apoptosis and enables survival and replication within the macrophage [10]. Indeed, recent studies have shown that virulent *M. tuberculosis* can subvert host apoptotic pathways by interfering with the production of key host signalling molecules [71], [73], resulting in the arrest of apoptosis and induction of necrosis, thereby offering an exit route from the macrophage [10]. Therefore, induction of anti-apoptotic genes as identified by the current study may provide another strategy used by the pathogen for the evasion of the host immune system.

### Induction of chemokine and cytokine genes following *M. bovis*-challenge

The innate immune response to mycobacterial pathogens is largely mediated by immuno-regulatory cytokines and chemokines secreted by various immune cells that regulate the recruitment and expansion of immune cell populations, such as monocytes, neutrophils, T cells and other effector cells from the blood to the site of infection [74]. Macrophage production of cytokines and chemokines in response to mycobacterial infection can be induced through a wide range of PRR-mediated pathways, and their secretion primes the adaptive immune response, which is pivotal in determining pathogenesis [75].

In the current study, several inflammatory chemokine and cytokine genes were shown to display large increases in relative expression following *M. bovis*-challenge across all of the time points analysed, with systems analysis showing that many of the top ranking GO categories were enriched for large numbers of these genes (see Tables S1, S2, S3, S4, S5, S6, S7, S8, S9, S10, S11, and S12). These results confirm that cytokine and chemokine gene expression is an early response of bovine macrophages to *M. bovis*-challenge following pathogen recognition by PRRs [14], [76].

Many of the upregulated inflammatory genes detected here have been shown to play an important role in the immune response to mycobacterial infection and several of these direct the transition from innate to adaptive immunity during infection [3]. Indeed, the GO categories identified via systems analysis of the differentially expressed genes here suggest that the expression of macrophage genes involved in the communication between innate and adaptive immune cell types is a key biological process that occurs within the first 24 hours of *M. bovis* challenge *in vitro*. For example, TNF-α is a key pleiotropic cytokine produced by macrophages that plays an important role in granuloma formation and maintenance by inducing IFN-γ release from T cells, which in turn, activates anti-mycobacterial function in infected macrophages [4], [77]. *IL12* (upregulated at all three time points) encodes a pro-inflammatory cytokine produced by macrophages that activates NK cells and T cells to produce IFN-γ and promote the adaptive immune response to mycobacterial infection [78]. IL-1β (encoded by *IL1B*, upregulated all time points) is a proinflammatory cytokine secreted largely by innate immune cells in response to mycobacterial infection and studies have shown that IL-1β is produced in excess at the site of infection, suggesting that it plays an important role in granuloma formation and maintenance [79]. Genes encoding β-chemokines (such as *CCL20*, which was upregulated at all three time points) participate in early immuno-protection against mycobacterial pathogens following infection [80], [81].

IL-6, a pleiotropic inflammatory cytokine expressed by a wide range of immune cells including macrophages in response to mycobacterial infection, has been proposed to play an important role in protection against tuberculosis [82]. *IL6* displayed large fold-upregulation at all three time points analysed in the *M. bovis*-challenged MDM, while the gene encoding the IL-6 receptor (*IL6R*) displayed downregulation expression in the *M. bovis*-challenged MDM at 2 hours and 24 hours and was not differentially expressed at the 6 hour time point. This suggests that endogenous IL-6 production by macrophages acts in an endocrine manner, presumably by initiating immune responses in other innate or adaptive immune cells [83]. Indeed, IL-6 has been proposed as a key regulator of the immunological switch between innate and adaptive immune processes [84].

Despite the role of chemokines and cytokines in directing the host immune response to control mycobacterial infection, there is evidence to suggest that their function can be used by mycobacterial pathogens to enable persistence within the host. For example, non-regulated production of TNF-α in lung tissue can result in immunopathology, including destructive inflammation and necrosis, allowing dissemination of the pathogen from infected cells [11], [85]. Furthermore, studies have shown that IL-6 can inhibit T cell responses following infection of macrophages with *M. bovis*-BCG and *M. avium* subspecies *paratuberculosis* [86], while other investigations have reported that *M. tuberculosis*-induced IL-6 production inhibits the anti-microbial activity of macrophages in response to IFN-γ [87]. *IL10*, which encodes an anti-inflammatory cytokine that limits local cytokine-induced tissue damage and systemic inflammatory responses during infection [88], was upregulated at 2 hours and 24 hours post-challenge. Interestingly, upregulation of *IL10* resulting in the subsequent suppression of host innate immune responses to infection has been proposed as a mechanism which enables enhanced mycobacterial intracellular proliferation [89]. Thus, exploitation of the roles of key pro- and anti-inflammatory cytokines may enable *M. bovis* to evade elimination by the host adaptive immune response, enabling disease progression.

### Conclusions

In the current study, we have analysed the bovine macrophage transcriptome in response to *M. bovis*-challenge *in vitro* using a global microarray platform. Inspection and systems analysis of the differentially expressed genes identified several key cellular pathways involved in the host response to *M. bovis*, including TLR and RLR signalling and apoptosis. These transcriptional signatures highlight the complex interactions between host macrophages and the mycobacterial pathogen during infection. Further analyses involving the comparison of gene expression changes in bovine macrophages to other mycobacterial species, such as *M. bovis*-BCG and *M. avium* subspecies *paratuberculosis*, may also highlight the differential responses of the host macrophage to these pathogens and enable identification of key cellular pathways that contribute to the development of pathology. Furthermore, the identification of gene expression patterns linked to infection and pathology could provide a route to improved BTB diagnostics.

## Supporting Information

Figure S1 **Regression analysis of relative gene expression fold-changes in the** ***M. bovis*** **-challenged MDM obtained from real time qRT-PCR analysis using conventionally-prepared and linearly amplified cDNA.** Log_2_ fold-changes in gene expression in the *M. bovis*-challenged MDM relative to the control MDM were plotted. Data obtained from the conventionally-prepared cDNA are plotted on the y-axis, while data obtained for the linearly amplified cDNA prepared using the WT-Ovation™ RNA Amplification System (OVA) are plotted on the x-axis. The *P*-value of the slope for all regression lines was≤0.001. Graph construction and analyses were performed using the Minitab version 16 software package. S = standard deviation; R-Sq = *r^2^* value.
(TIF)

Figure S2 **Comparison of the fold-changes in expression for the** ***CCL5*** **, CCL20,** ***IL1B*** **and** ***IL6*** **genes based on real time qRT-PCR analysis using conventional and linearly amplified cDNA.** Log_2_ fold-changes in expression in the *M. bovis*-challenged MDM relative to the control MDM at all three time points are shown. Linearly amplified cDNA template (OVA) was prepared using the WT-Ovation™ RNA Amplification System. For comparison, the expression profiles for these genes as per the microarray data are also shown. The significance of the fold-changes in expression for each gene based on the real time qRT-PCR analysis only are denoted by asterisks in the figure (*P≤0.05, **P≤0.01, ***P≤0.001). The fold-changes calculated for each gene the microarray data in the *M. bovis*-challenged MDM for each gene were significant (adjusted *P*-value≤0.05). In addition, the log_2_ fold-change in expression for the control MDM at each time point relative to the 0 hour control MDM are also shown for both the microarray and real time qRT-PCR data; no significant differences in gene expression between the control MDM relative to the 0 hour control was observed at each time point (*P*≥0.05).
(PDF)

Figure S3 **Real time qRT-PCR analysis.** Log_2_ fold-changes in expression in the *M. bovis*-challenged MDM relative to the control MDM at all three time points are shown. For comparison, the expression profiles for these genes as per the microarray data are also shown. The significance of the mean fold-changes in expression for each gene based on the real time qRT-PCR analysis only are denoted by asterisks in the figure (*P≤0.05, **P≤0.01, ***P≤0.001). The mean fold-changes calculated for each gene based on the microarray data in the *M. bovis*-challenged MDM for each gene were significant (adjusted *P*-value≤0.05)―probes for *TICAM1* were not present on the microarray and this gene was analysed by real time qRT-PCR only. In addition, the log_2_ fold-change in expression for the control MDM at each time point relative to the 0 hour control MDM are also shown for both the microarray and real time qRT-PCR data; no significant differences in gene expression between the control MDM relative to the 0 hour control was observed at each time point (*P*≥0.05).
(PDF)

Table S1 **Real time qRT-PCR primers used in this study.**
(DOC)

Table S2 **The list of differentially expressed transcripts detected at the 2 hour time point post-** ***M. bovis*** **challenge.**
(XLS)

Table S3 **Gene ontology (GO) categories identified using IPA at the 2 hour time point post-** ***M. bovis*** **challenge.** The top ranking GO categories identified by IPA are listed according to *P*-values.
(XLS)

Table S4 **Top-ranking canonical pathways identified using IPA at the 2 hour time point post-** ***M. bovis*** **challenge.** The top ranking canonical pathways identified by IPA are listed according to *P*-values. The ratio indicates the number of differentially expressed genes involved in each canonical pathway divided by the total number of genes within each pathway as per the IPA Knowledge Base.
(XLS)

Table S5 **The list of differentially expressed transcripts detected at the 6 hour time point post-** ***M. bovis*** **challenge.**
(XLS)

Table S6 **Gene ontology (GO) categories identified using IPA at the 6 hour time point post-** ***M. bovis*** **challenge.** The top ranking GO categories identified by IPA are listed according to *P*-values.
(XLS)

Table S7 **Top-ranking canonical pathways identified using IPA at the 6 hour time point post-** ***M. bovis*** **challenge.** The top ranking canonical pathways identified by IPA are listed according to *P*-values. The ratio indicates the number of differentially expressed genes involved in each canonical pathway divided by the total number of genes within each pathway according to the IPA Knowledge Base.
(XLS)

Table S8 **The list of differentially expressed transcripts detected at the 24 hour time point post-** ***M. bovis*** **challenge.**
(XLS)

Table S9 **Gene ontology (GO) categories identified using IPA at the 24 hour time point post-** ***M. bovis*** **challenge.** The top ranking GO categories identified by IPA are listed according to *P*-values.
(XLS)

Table S10 **Top-ranking canonical pathways identified using IPA at the 24 hour time point post-** ***M. bovis*** **challenge.** The top ranking canonical pathways identified by IPA are listed according to *P*-values. The ratio indicates the number of differentially expressed genes involved in each canonical pathway divided by the total number of genes within each pathway according to the IPA Knowledge Base.
(XLS)

Table S11 **Comparison of relative gene expression fold-changes in the** ***M. bovis*** **-challenged MDM obtained from real time qRT-PCR analysis using conventionally-prepared and linearly amplified cDNA.** Geometric mean fold-changes in gene expression (*M. bovis*-challenged MDM versus control MDM) are given. *P*-values were obtained by statistical analysis of log_2_ fold-change gene expression data.
(DOC)

Table S12 **Comparison of fold-changes in gene expression in the** ***M. bovis*** **-challenged MDM based on microarray and real time qRT-PCR results.** Geometric mean fold-changes of gene expression (*M. bovis*-challenged MDM relative to control MDM) are given for the microarray and real time qRT-PCR data at each time point. All genes at each time point were significantly differentially expressed based on the microarray data (adjusted *P*-value≤0.05). The *P*-values obtained from analysis of the real time qRT-PCR analysis are presented. Descriptions of the function of each gene were obtained from the GeneCards version 3 database [90]. ‘Not DE’ indicates that the gene was note differentially expressed according to the real time qRT-PCR data.
(DOC)
