# Supplementary figures and images for: Global Gene Expression and Systems Biology Analysis of Bovine Monocyte-Derived Macrophages in Response to In Vitro Challenge with Mycobacterium bovis
Source: PLoS One. 2012 Feb 22;7(2):e32034. doi: 10.1371/journal.pone.0032034 (PMC3284544; doi:10.1371/journal.pone.0032034)

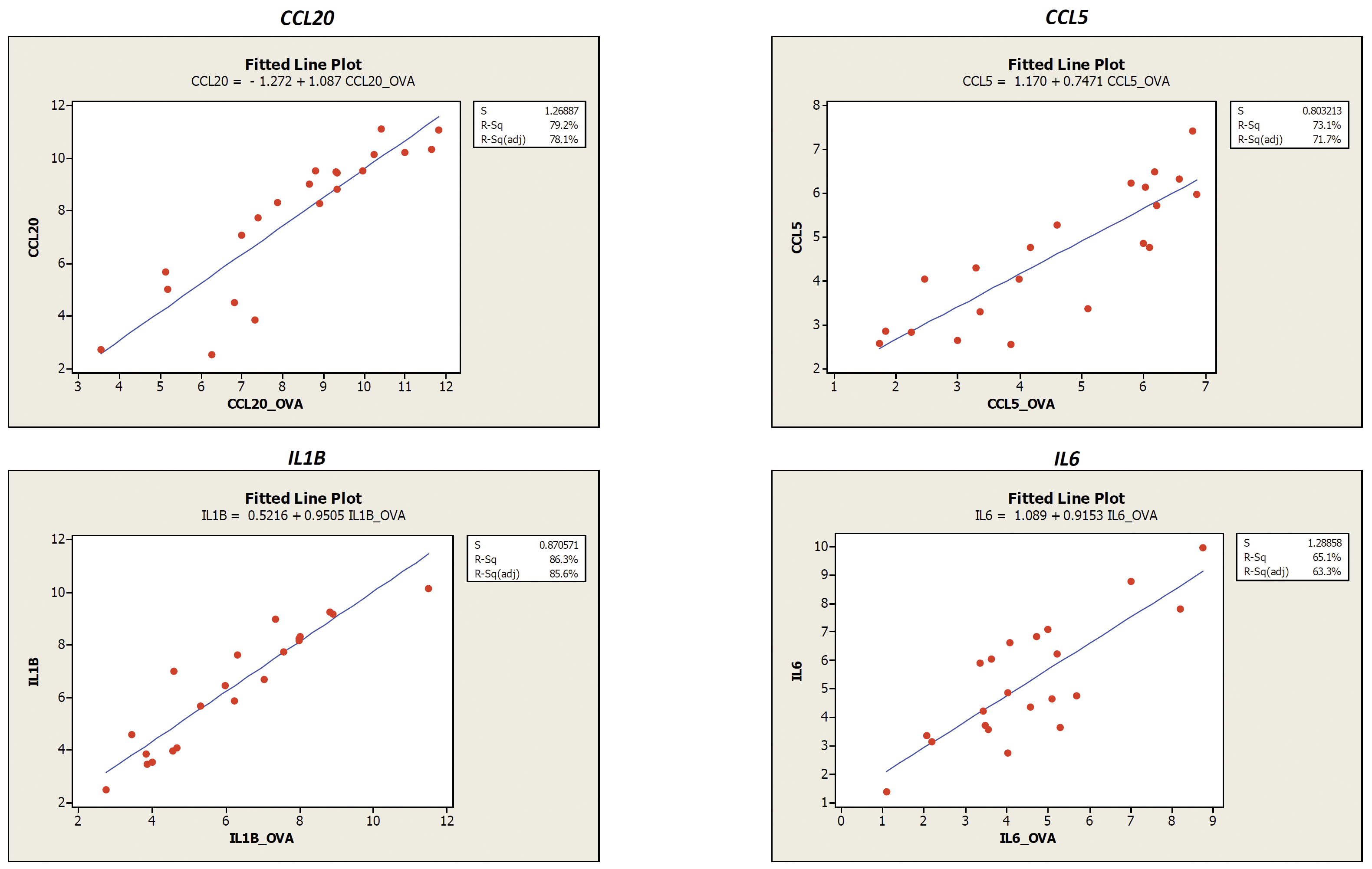

Supplement: Figure S1 — Regression analysis of relative gene expression fold-changes in the M. bovis -challenged MDM obtained from real time qRT-PCR analysis using conventionally-prepared and linearly amplified cDNA. Log2 fold-changes in gene expression in the M. bovis-challenged MDM relative to the control MDM were plotted. Data obtained from the conventionally-prepared cDNA are plotted on the y-axis, while data obtained for the linearly amplified cDNA prepared using the WT-Ovation™ RNA Amplification System (OVA) are plotted on the x-axis. The P-value of the slope for all regression lines was≤0.001. Graph construction and analyses were performed using the Minitab version 16 software package. S = standard deviation; R-Sq = r2 value. (TIF) [file pone.0032034.s001.tif]

*CCL5*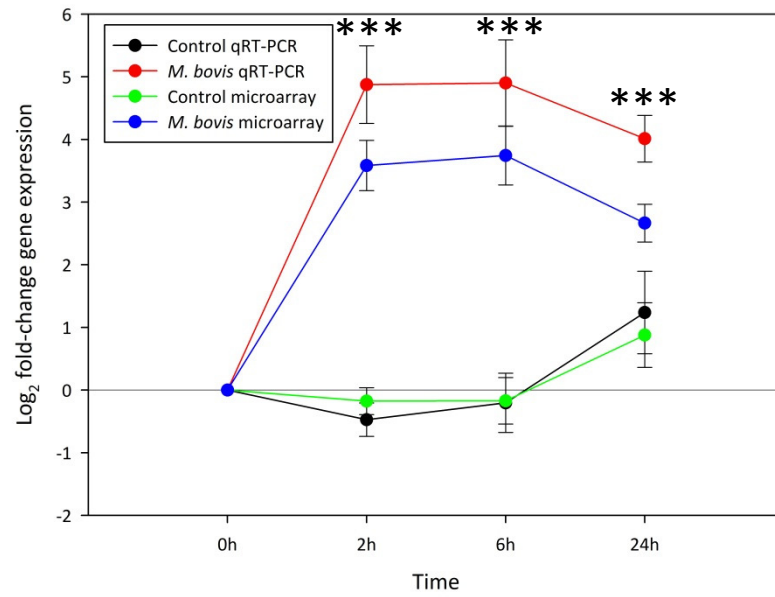*CCL5\_OVA*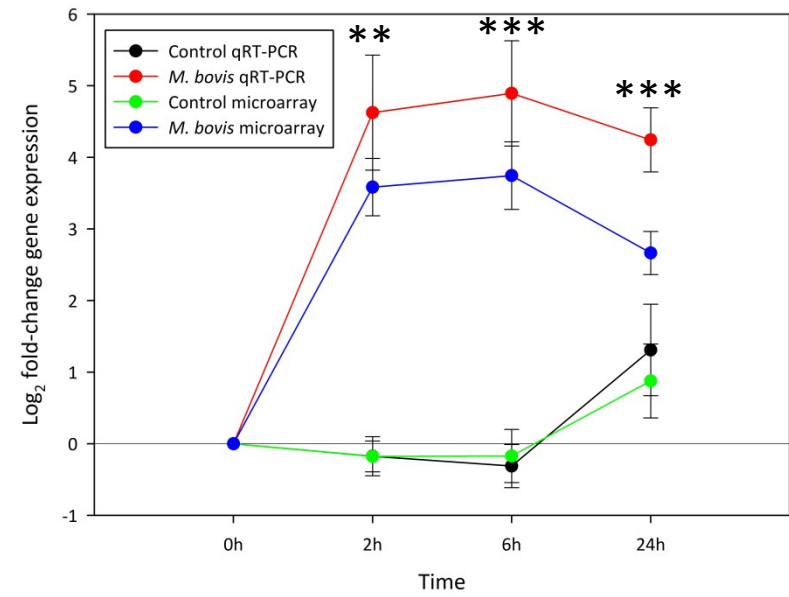*CCL20*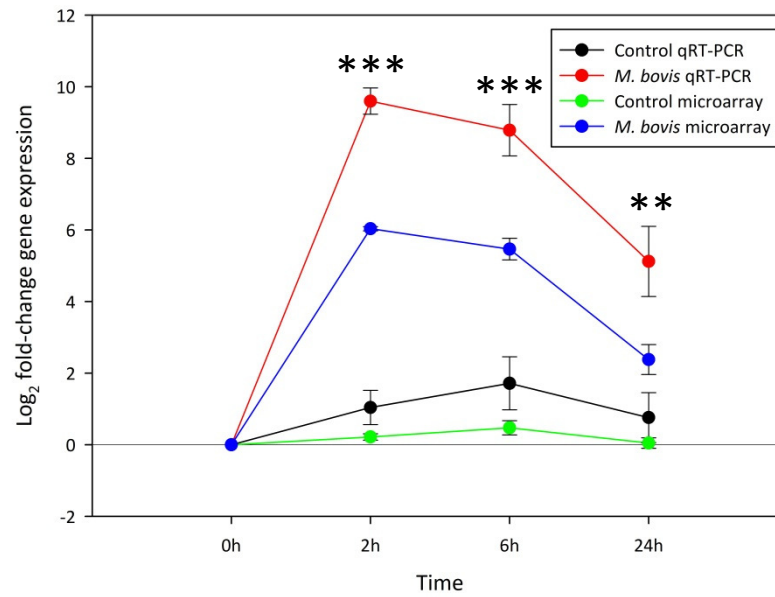*CCL20\_OVA*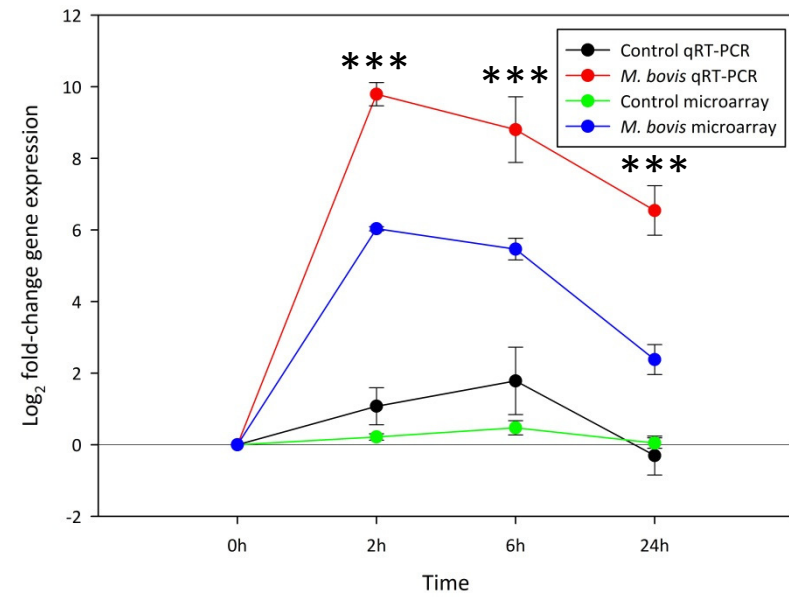

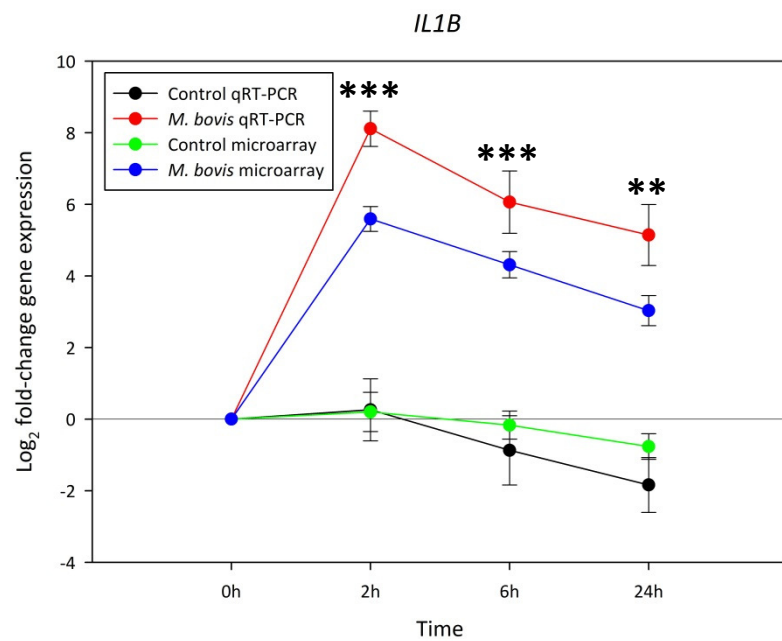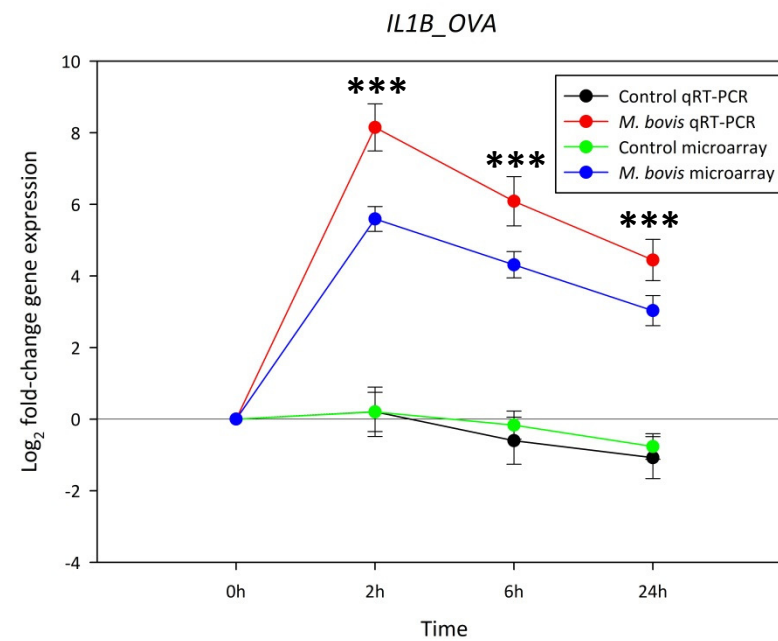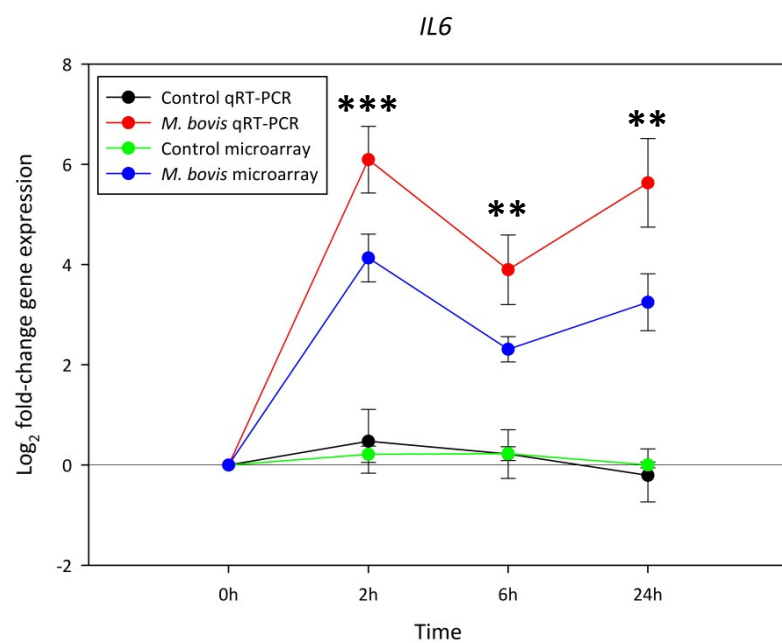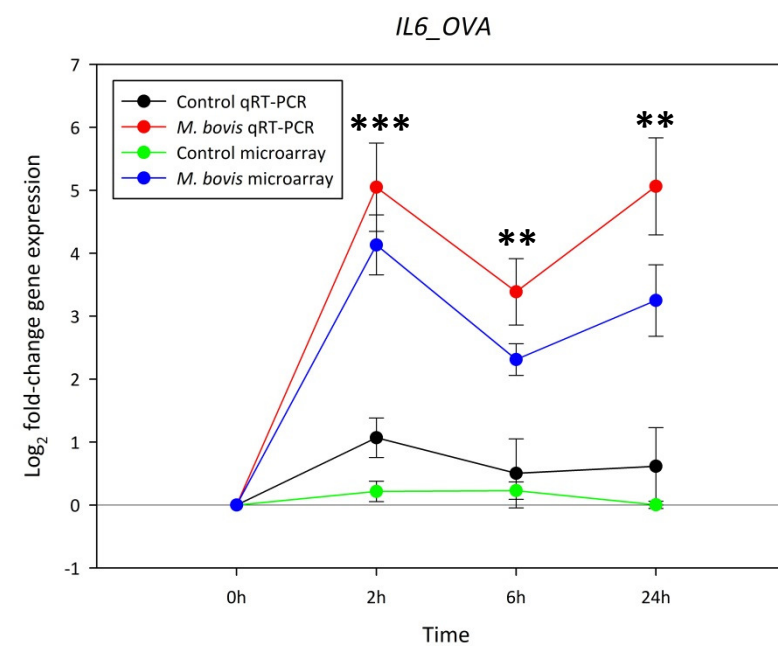

Supplement: Figure S2 — Comparison of the fold-changes in expression for the CCL5 , CCL20, IL1B and IL6 genes based on real time qRT-PCR analysis using conventional and linearly amplified cDNA. Log2 fold-changes in expression in the M. bovis-challenged MDM relative to the control MDM at all three time points are shown. Linearly amplified cDNA template (OVA) was prepared using the WT-Ovation™ RNA Amplification System. For comparison, the expression profiles for these genes as per the microarray data are also shown. The significance of the fold-changes in expression for each gene based on the real time qRT-PCR analysis only are denoted by asterisks in the figure (*P≤0.05, **P≤0.01, ***P≤0.001). The fold-changes calculated for each gene the microarray data in the M. bovis-challenged MDM for each gene were significant (adjusted P-value≤0.05). In addition, the log2 fold-change in expression for the control MDM at each time point relative to the 0 hour control MDM are also shown for both the microarray and real time qRT-PCR data; no significant differences in gene expression between the control MDM relative to the 0 hour control was observed at each time point (P≥0.05). (PDF) [file pone.0032034.s002.pdf]

*AREGB*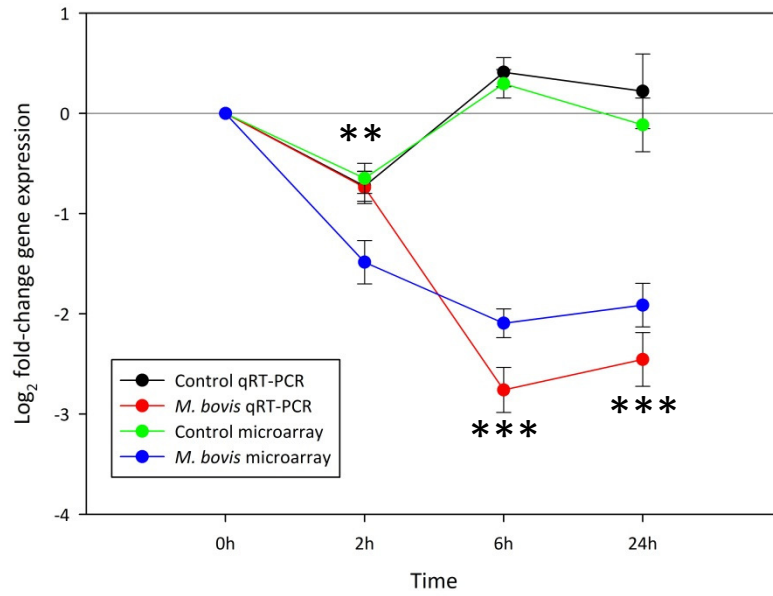*CCL4*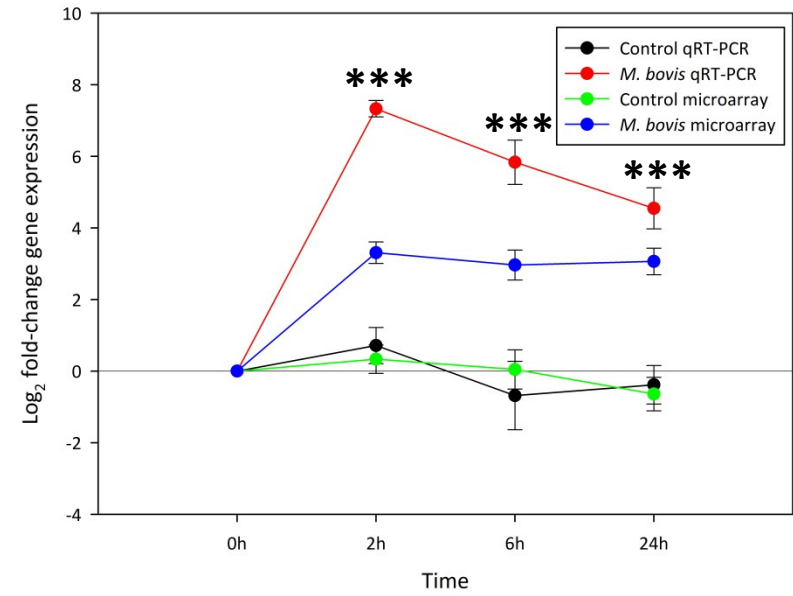*CCL5*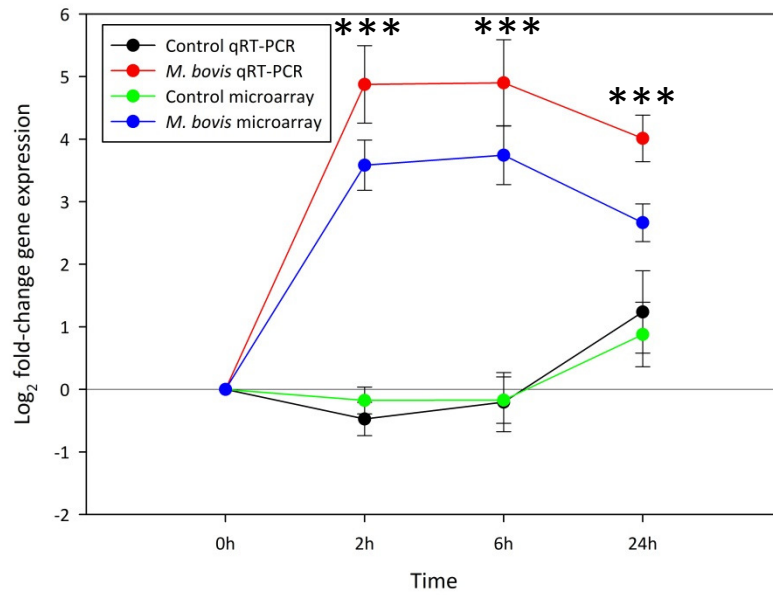*CCL20*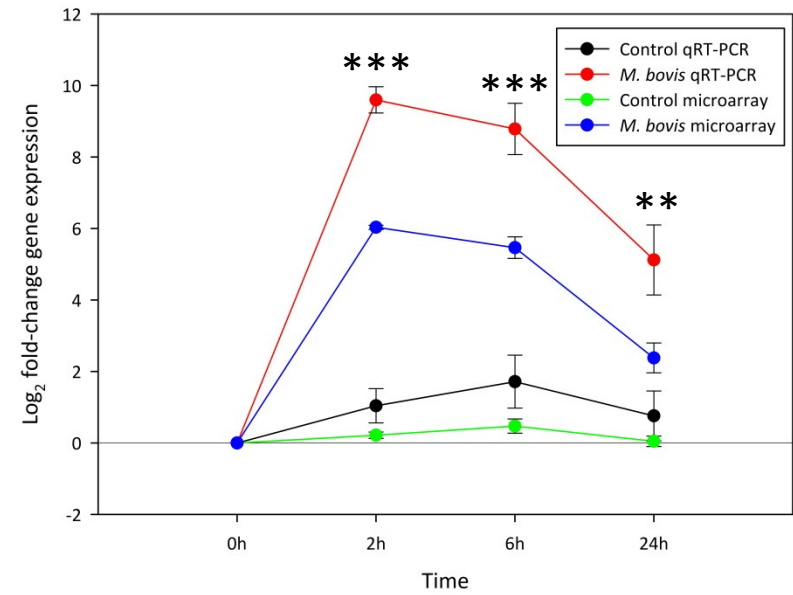

CD40

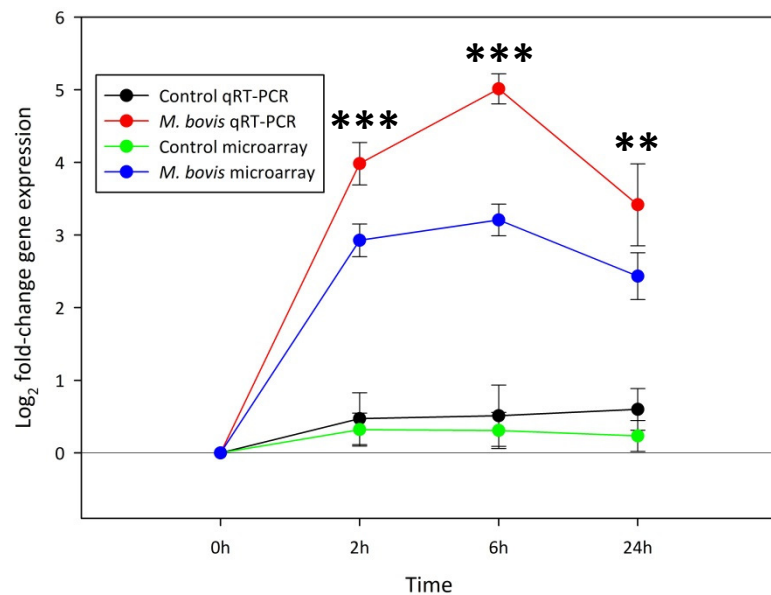

CFB

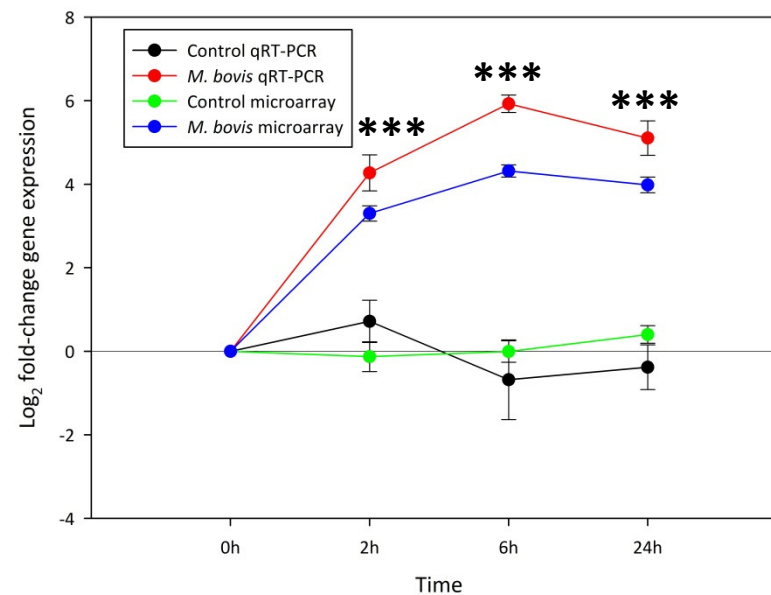

CXCL2

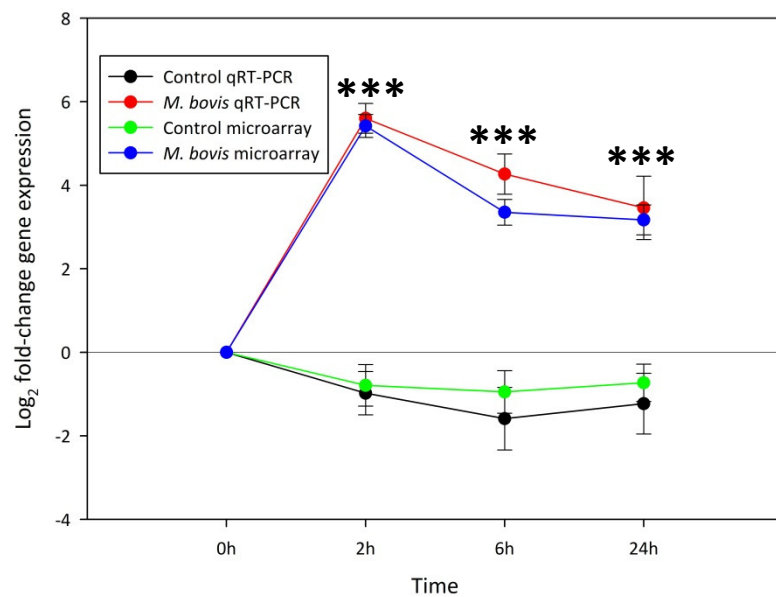

FOS

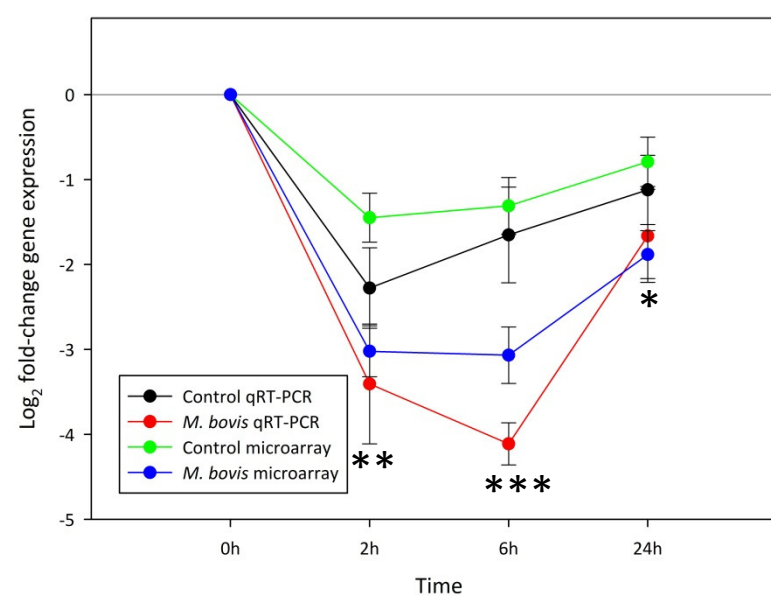

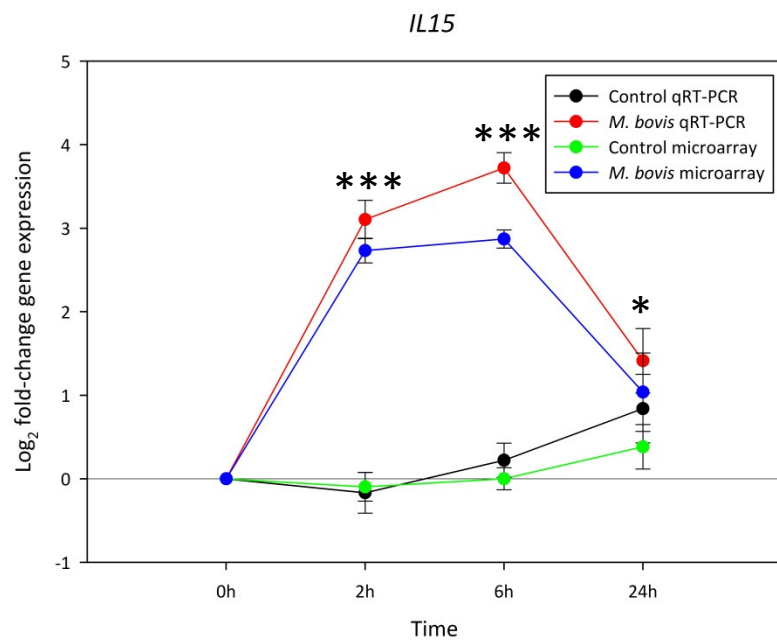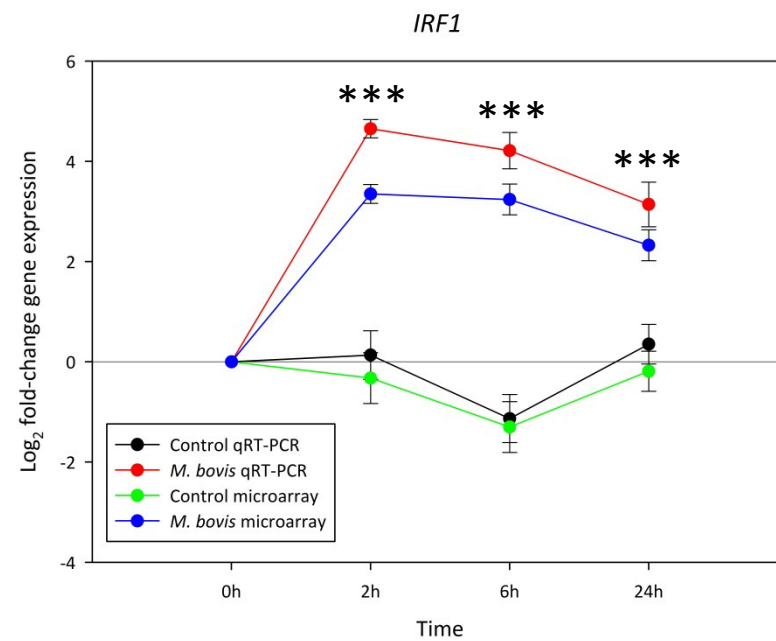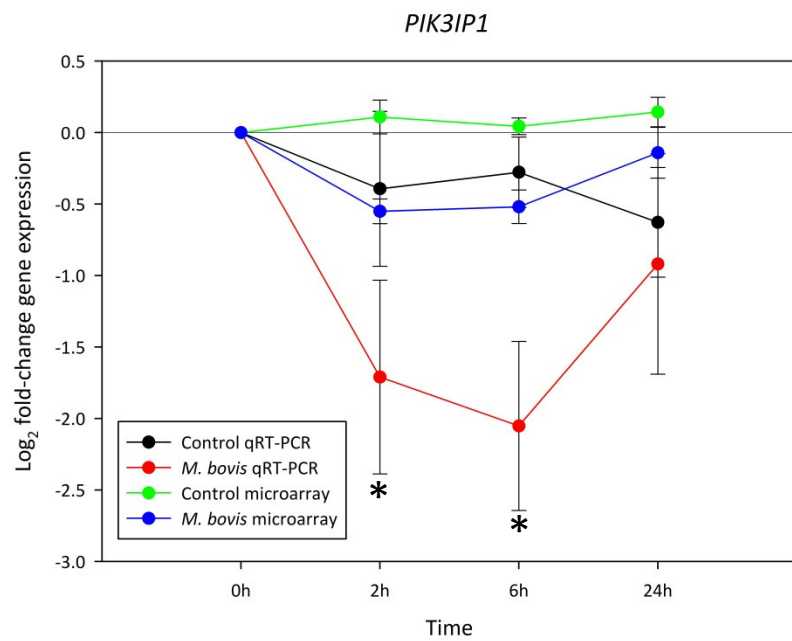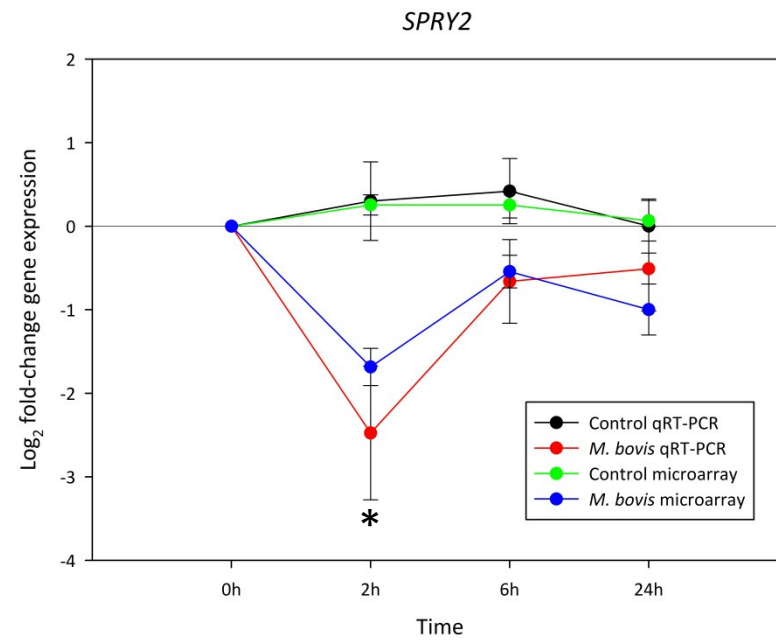

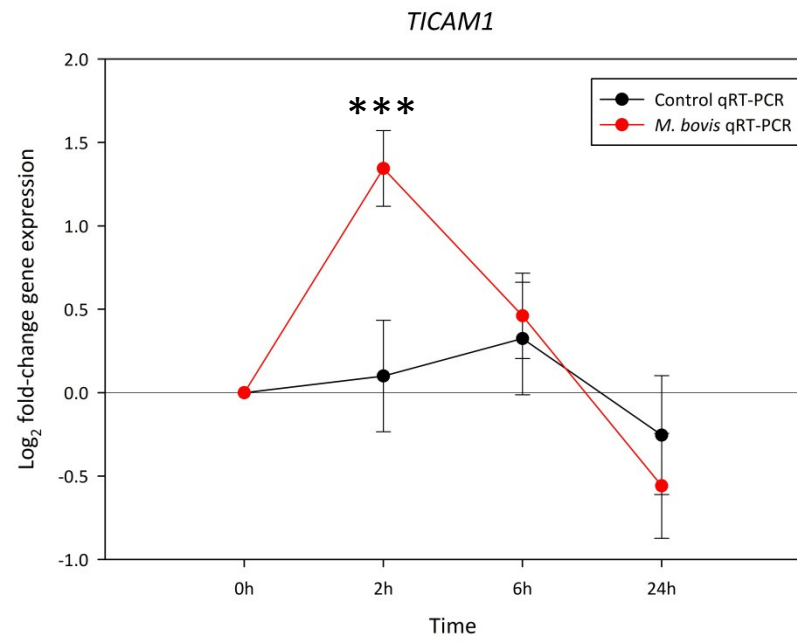

Supplement: Figure S3 — Real time qRT-PCR analysis. Log2 fold-changes in expression in the M. bovis-challenged MDM relative to the control MDM at all three time points are shown. For comparison, the expression profiles for these genes as per the microarray data are also shown. The significance of the mean fold-changes in expression for each gene based on the real time qRT-PCR analysis only are denoted by asterisks in the figure (*P≤0.05, **P≤0.01, ***P≤0.001). The mean fold-changes calculated for each gene based on the microarray data in the M. bovis-challenged MDM for each gene were significant (adjusted P-value≤0.05)―probes for TICAM1 were not present on the microarray and this gene was analysed by real time qRT-PCR only. In addition, the log2 fold-change in expression for the control MDM at each time point relative to the 0 hour control MDM are also shown for both the microarray and real time qRT-PCR data; no significant differences in gene expression between the control MDM relative to the 0 hour control was observed at each time point (P≥0.05). (PDF) [file pone.0032034.s003.pdf]
