# Supplementary material for: Global Gene Expression and Systems Biology Analysis of Bovine Monocyte-Derived Macrophages in Response to In Vitro Challenge with Mycobacterium bovis
Source: PLoS One. 2012 Feb 22;7(2):e32034. doi: 10.1371/journal.pone.0032034 (PMC3284544; doi:10.1371/journal.pone.0032034)
Supplement: Table S1 — Real time qRT-PCR primers used in this study. (DOC) [file pone.0032034.s004.doc]

**Table S1. R**eal time qRT-PCR primers used in this study

| **Gene name** | **Gene Symbol** | **Ensembl database ID** | **Forward Primer Sequence (5′-3′)** | **Reverse Primer Sequence (5′-3′)** | **Amplicon size (bp)** | **Exon spanning** |
| --- | --- | --- | --- | --- | --- | --- |
| Amphiregulin B | *AREGB* | ENSBTAG00000018134 | CGTCTCTGCCATGACCTTCA | TTTCGTTCTTCAGCGACACC | 99 | 5-6 |
| Chemokine (C-C motif) ligand 4 | *CCL4* | ENSBTAG00000025257 | AGCTGTGGTATTCCAGACCAA | TCAAGGTCATCCACGTACTCC | 87 | 2-3 |
| Chemokine (C-C motif) ligand 5 | *CCL5* | ENSBTAG00000007191 | AGCAGTTGTCTTTATCACCAGGA | TCCAAAGCGTTGATGTACTCTC | 87 | 2-3 |
| Chemokine (C-C motif) ligand 20 | *CCL20* | ENSBTAG00000021326 | AATTAGCTGTGTGTGCAGATCC | CATCCTTTTGACTCTTTGACTGA | 80 | 3-4 |
| CD40 molecule, TNF receptor superfamily member 5 | *CD40* | ENSBTAG00000020736 | TCCTGTTTGCTGTCCTGTTG | GATCCTGCCTTTCAGCCATA | 96 | 7-9 |
| Complement factor B | *CFB* | ENSBTAG00000007450 | CTTCATTCAAGTTGGCGTGA | GCAGCACCTGGTAGAGGTTG | 110 | 17-18 |
| Chemokine (C-X-C motif) ligand 2 | *CXCL2* | ENSBTAG00000037558 | TGGTCAGGAAGTGTGTCTCAA | TCAGTTGGCACTAGCCTTGTT | 85 | 3-4 |
| FBJ murine osteosarcoma viral oncogene homolog | *FOS* | ENSBTAG00000004322 | CAAGCGGAGACAGACCAACT | TGAGCCGCTAGGATGAACTC | 104 | 3-4 |
| Interleukin 15 | *IL15* | ENSBTAG00000018200 | ACCATGCTAGCAAACAGCAA | TTCCTCCAGTTCCTCACATTC | 81 | 5-6 |
| Interleukin 1, beta | *IL1B* | ENSBTAG00000001321 | ACCTGAACCCATCAACGAAATG | TAGGGTCATCAGCCTCAAATAACA | 74 | 2-3 |
| Interleukin 6 | *IL6* | ENSBTAG00000014921 | ATCAGAACACTGATCCAGATCC | CAAGGTTTCTCAGGATGAGG | 145 | 4-5 |
| Interferon regulatory factor 1 | *IRF1* | ENSBTAG00000031231 | GCCCACCTCTGTCTATGGAG | CAGCTGGGATCCATGTTCTT | 111 | 9-10 |

**Table S1 continued. The real time qRT-PCR primers used in this study**

| **Gene name** | **Gene Symbol** | **Ensembl database ID** | **Forward Primer Sequence (5′-3′)** | **Reverse Primer Sequence (5′-3′)** | **Amplicon size (bp)** | **Exon spanning** |
| --- | --- | --- | --- | --- | --- | --- |
| Nuclear factor of kappa light polypeptide gene enhancer in B-cells 2 (p49/p100) | *NFKB2* | ENSBTAG00000006017 | CCTGCTGAATGCTCTGTCTG | TCCTCCTTCACCTCTGTGCT | 102 | 23-24 |
| Phosphoinositide-3-kinase interacting protein 1 | *PIK3IP1* | ENSBTAG00000010667 | GGAGCTGGAATTGTCCTTGG | GCACACTTTCTGCTCGTGCT | 75 | 5-6 |
| Peptidylprolyl isomerase A (cyclophilin A) | *PPIA* | ENSBTAG00000012003 | CATACAGGTCCTGGCATCTTGTCC | CACGTGCTTGCCATCCAACC | 108 | 4-5 |
| Sprouty homolog 2 (*Drosophila*) | *SPRY2* | ENSBTAG00000001774 | AGCCAAGGGTTGCCTTAAAT | GTGGGACAGTGGGAACTTTG | 110 | Only 1 exon |
| Toll-like receptor adaptor molecule 1 | *TICAM1* | ENSBTAG00000019966 | TGCTCTAGACCACTCGGCATT | CAGGCGGCAGTCAAAGTTG | 61 | 1-2 |
| Tumor necrosis factor (TNF superfamily, member 2) | *TNF* | ENSBTAG00000025471 | GCTCCAGAAGTTGCTTGTGC | AACCAGAGGGCTGTTGATGG | 149 | 1-2 |
