# Supplementary material for: Global Gene Expression and Systems Biology Analysis of Bovine Monocyte-Derived Macrophages in Response to In Vitro Challenge with Mycobacterium bovis
Source: PLoS One. 2012 Feb 22;7(2):e32034. doi: 10.1371/journal.pone.0032034 (PMC3284544; doi:10.1371/journal.pone.0032034)
Supplement: Table S11 — Comparison of relative gene expression fold-changes in the M. bovis -challenged MDM obtained from real time qRT-PCR analysis using conventionally-prepared and linearly amplified cDNA. Geometric mean fold-changes in gene expression (M. bovis-challenged MDM versus control MDM) are given. P-values were obtained by statistical analysis of log2 fold-change gene expression data. (DOC) [file pone.0032034.s014.doc]

**Table S11: Comparison of relative gene expression fold-changes in the *M. bovis*-challenged MDM obtained from real time qRT-PCR analysis using conventionally-prepared and linearly amplified cDNA**

| **Time** | **2 hours** | | | | **6 hours** | | | | **24 hours** | | | |
| --- | --- | --- | --- | --- | --- | --- | --- | --- | --- | --- | --- | --- |
| **cDNA template** | **Conventional cDNA** | | **Amplified cDNA** | | **Conventional cDNA** | | **Amplified cDNA** | | **Conventional cDNA** | | **Amplified cDNA** | |
| **Gene** | **Mean fold-change** | ***P*-value** | **Mean fold-change** | ***P*-value** | **Mean fold-change** | ***P*-value** | **Mean fold-change** | ***P*-value** | **Mean fold-change** | ***P*-value** | **Mean fold-change** | ***P*-value** |
| *CCL5* | +29.36 | ≤ 0.001 | +24.68 | ≤ 0.01 | +29.87 | ≤ 0.001 | +29.72 | ≤ 0.01 | 16.14 | ≤ 0.001 | 18.95 | ≤ 0.001 |
| *CCL20* | +775.56 | ≤ 0.001 | +887.65 | ≤ 0.001 | +441.13 | ≤ 0.001 | +446.48 | ≤ 0.001 | 34.81 | ≤ 0.01 | 93.34 | ≤ 0.001 |
| *IL1B* | +276.96 | ≤ 0.001 | +283.87 | ≤ 0.001 | +66.89 | ≤ 0.001 | +67.93 | ≤ 0.001 | 35.38 | ≤ 0.001 | 21.80 | ≤ 0.001 |
| *IL6* | +68.33 | ≤ 0.001 | +33.12 | ≤ 0.001 | +14.91 | ≤ 0.001 | +10.47 | ≤ 0.01 | 49.61 | ≤ 0.001 | 33.45 | ≤ 0.01 |
