# Supplementary material for: Global Gene Expression and Systems Biology Analysis of Bovine Monocyte-Derived Macrophages in Response to In Vitro Challenge with Mycobacterium bovis
Source: PLoS One. 2012 Feb 22;7(2):e32034. doi: 10.1371/journal.pone.0032034 (PMC3284544; doi:10.1371/journal.pone.0032034)
Supplement: Table S12 — Comparison of fold-changes in gene expression in the M. bovis -challenged MDM based on microarray and real time qRT-PCR results. Geometric mean fold-changes of gene expression (M. bovis-challenged MDM relative to control MDM) are given for the microarray and real time qRT-PCR data at each time point. All genes at each time point were significantly differentially expressed based on the microarray data (adjusted P-value≤0.05). The P-values obtained from analysis of the real time qRT-PCR analysis are presented. Descriptions of the function of each gene were obtained from the GeneCards version 3 database [90]. ‘Not DE’ indicates that the gene was note differentially expressed according to the real time qRT-PCR data. (DOC) [file pone.0032034.s015.doc]

**Table S12: Comparison of fold-changes in gene expression in the *M. bovis*-challenged MDM based on microarray and real time qRT-PCR results**

| **Gene symbol** | **Gene name** | **Gene description** | **2 hour mean fold-changes in gene expression** | | | **6 hour mean fold-changes in gene expression** | | | **24 hour mean fold-changes in gene expression** | | |
| --- | --- | --- | --- | --- | --- | --- | --- | --- | --- | --- | --- |
|  |  |  | **Microarray data** | **Real time qRT-PCR** | ***P*-value** | **Microarray data** | **Real time qRT-PCR** | ***P*-value** | **Microarray data** | **Real time qRT-PCR** | ***P*-value** |
| *AREGB* | Amphiregulin B | A growth-modulating glycoprotein | -1.79 | -1.67 | ≤ 0.01 | -5.24 | -6.77 | ≤ 0.001 | -3.48 | -5.48 | ≤ 0.001 |
| *CCL4* | Chemokine (C-C motif) ligand 4 | A proinflammatory and chemotactic chemokine | +9.92 | +161.10 | ≤ 0.001 | +7.81 | +57.09 | ≤ 0.001 | +8.37 | 23.39 | ≤ 0.001 |
| *CCL5* | Chemokine (C-C motif) ligand 5 | A proinflammatory chemokine involved in the chemotaxis of monocytes and T-helper cells | +12.00 | +29.36 | ≤ 0.001 | +13.41 | +29.87 | ≤ 0.001 | +6.34 | 16.14 | ≤ 0.001 |
| *CCL20* | Chemokine (C-C motif) ligand 20 | A chemokine involved in the chemoattraction of lymphocytes and neutrophils | +65.53 | +775.56 | ≤ 0.001 | +44.18 | +441.13 | ≤ 0.001 | +5.20 | 34.81 | ≤ 0.01 |
| *CD40* | CD40 molecule, TNF receptor superfamily member 5 | A member of the TNF-receptor superfamily; mediates the immune and inflammatory responses | +7.61 | +15.82 | ≤ 0.001 | +9.25 | +32.30 | ≤ 0.001 | +5.41 | 10.68 | ≤ 0.001 |
| *CFB* | Complement factor B | A component of the alternative pathway of complement activation | +10.78 | +19.33 | ≤ 0.001 | +20.02 | +60.87 | ≤ 0.001 | +11.97 | 34.40 | ≤ 0.001 |
| *CXCL2* | Chemokine (C-X-C motif) ligand 2 | An immunoregulatory chemokine produced by activated monocytes and neutrophils at sites of inflammation | +25.57 | +48.57 | ≤ 0.001 | +11.76 | +19.27 | ≤ 0.001 | +7.08 | 10.99 | ≤ 0.01 |
| *FOS* | FBJ murine osteosarcoma viral oncogene homolog | A leucine zipper protein member of the AP-1 transcription factor complex | -4.19 | -10.60 | ≤ 0.01 | -4.94 | -17.28 | ≤ 0.001 | -2.24 | -3.17 | ≤ 0.05 |
| *IL1B* | Interleukin 1, beta | A cytokine that mediates the inflammatory response including cell proliferation, differentiation and apoptosis | +35.52 | +276.96 | ≤ 0.001 | +17.96 | +66.89 | ≤ 0.001 | +9.85 | 35.38 | ≤ 0.001 |
| *IL6* | Interleukin 6 | A cytokine that functions in inflammation and the maturation of B cells | +17.54 | +68.33 | ≤ 0.001 | +4.96 | +14.91 | ≤ 0.001 | +9.51 | 49.61 | ≤ 0.001 |
| *IL15* | Interleukin 15 | A cytokine that regulates T and natural killer cell activation and proliferation | +6.65 | +8.60 | ≤ 0.001 | +7.32 | +13.19 | ≤ 0.001 | +2.05 | 2.66 | ≤ 0.05 |
| *IRF1* | Interferon regulatory factor 1 | A member of the interferon regulatory transcription factor family; an activator of interferon alpha and beta transcription | +10.86 | +25.15 | ≤ 0.001 | +9.82 | +18.57 | ≤ 0.001 | +5.09 | 8.83 | ≤ 0.001 |
| *NFKB2* | Nuclear factor of kappa light polypeptide gene enhancer in B-cells 2 (p49/p100) | A pleiotropic transcription factor involved in inflammation, immunity, differentiation, cell growth and apoptosis | +5.34 | +8.00 | ≤ 0.001 | +8.16 | +13.15 | ≤ 0.001 | +3.37 | 4.92 | ≤ 0.01 |
| *PIK3IP1* | Phosphoinositide-3-kinase interacting protein 1 | Suppresses the activity of phosphatidylinositol-3-kinase (PI3K); a regulator of cell division | -2.50 | -3.27 | ≤ 0.05 | -2.02 | -4.15 | ≤ 0.05 | -1.39 | Not DE | ≥ 0.05 |
| *SPRY2* | Sprouty homolog 2 (*Drosophila*) | An inhibitor of receptor tyrosine kinase signalling proteins | -3.84 | -5.56 | ≤ 0.05 | -1.74 | Not DE | ≥ 0.05 | -2.09 | Not DE | ≥ 0.05 |
| *TICAM1* | Toll-like receptor adaptor molecule 1 | An adapter protein used by TLR3 and TLR4 (through TICAM2) to mediate NF-κB and interferon-regulatory factor (IRF) activation | N/A | +2.54 | ≤ 0.001 | N/A | Not DE | ≥ 0.05 | N/A | Not DE | ≥ 0.05 |
| *TNF* | Tumor necrosis factor (TNF superfamily, member 2) | A proinflammatory cytokine secreted by macrophages involved in the regulation of cell proliferation, differentiation and apoptosis | +31.87 | +93.52 | ≤ 0.001 | +16.23 | +47.02 | ≤ 0.001 | +5.23 | 14.09 | ≤ 0.001 |
